# Biodiversity management of organic orchard enhances both ecological and economic profitability

**DOI:** 10.7717/peerj.2137

**Published:** 2016-06-23

**Authors:** Jie Meng, Lijun Li, Haitao Liu, Yong Li, Caihong Li, Guanglei Wu, Xiaofan Yu, Liyue Guo, Da Cheng, Mahmud A. Muminov, Xiaotian Liang, Gaoming Jiang

**Affiliations:** 1 State Key Laboratory of Vegetation and Environment Change, Institute of Botany, Chinese Academy of Sciences, Beijing, China; 2 University of Chinese Academy of Sciences, Beijing, China; 3 Key Laboratory of Ecosystem Network Observation and Modeling, Institute of Geographic Sciences and Natural Resources Research, Chinese Academy of Sciences, Beijing, China

**Keywords:** Agroecology, Organic apple orchard, Biodiversity management, Soil bacterial diversity, 16S rDNA, Weed control, Pest control, Earthworms, Eco-economic benefits

## Abstract

Organic farming has been regarded as an alternative solution for both agricultural sustainability and human health maintenance. Few researches have concentrated on the differences of biodiversity and eco-economic benefits between organic and conventional orchards. Organic management (OM) of orchards mainly includes taking advantage of natural enemies and beneficial weeds as well as soil organisms and controlling harmful pests. Here we conducted a three-year experiment on the effects of managing biodiversity in an organic apple orchard, using cattle manure to enrich soil biota, propagating native plant to suppress weeds and applying ecological pest management to control pests. The effect was assessed against the conventional management (CM) model. We found that OM enhanced soil organic carbon, total nitrogen, microbial biomass carbon and nitrogen. The 16S rDNA high-throughput sequencing results indicated that the dominant bacterial phyla of the top soil were *Proteobacteria* and *Actinobacteria*, and OM had richer bacteria diversity with a 7% higher Shannon’s index than the CM. In particular, the relative abundance of rhizobium in the OM was higher than that of the CM. For OM, *Duchesnea indica* was an ideal ground-cover plant to control weeds through winning the niche competition and thus decreased weeds’ Simpson, Shannon–Wiener and Pielou index by 38.2%, 53.8% and 16.9% separately. The phototactic pests’ weight and scarab beetle’s population were effectively decreased by 35% and 86% respectively through long time control and prevention. OM had an average of 20 times more earthworms than CM, and the maximum density had reached 369 m^−2^ (0–20 cm soil). The dominant earthworm species of the OM were detritivores which preferring soil with high organic matter content. Due to no synthetic chemicals being used, the OM produced much safer apple fruits which were sold at high prices. Economically, up to a 103% increase of output–input ratio had been achieved in the OM. Our study clearly demonstrated that biodiversity management without chemical pollution increased the biodiversity of beneficial organisms, reduced antagonists of the fruit tree, and enhanced economic benefits of the apple orchard.

## Introduction

Increasing usage of synthetic fertilizers, pesticides and herbicides in conventional farming has proved to be a tremendous threat to food safety and to the ecosystem’s health and sustainability (Shorette, 2012). Consequent problems such as soil acidification (Kibblewhite, Ritz & Swift, 2008), soil infertility as well as soil, water and atmospheric contamination have caused continuing deterioration (Guo et al., 2010; Tian & Niu, 2015). The biodiversity of plant and microbe has also declined (Robinson & Sutherland, 2002; Yu et al., 2015) because of the above mentioned problems with losing balance of the agroecosystem. Even worse, as chemical pesticides and herbicides have high eco-toxicity and synergistic toxicity (Laetz et al., 2009), pesticides and the residues that are frequently found in foods are devastating human health through different toxic mechanisms such as poisoning of gastrointestinal, renal, nervous systems and pulmonary fibrosis (Eddleston & Bateman, 2012; Margni et al., 2002). To avoid those crises, people begin to rethink whether or not the yield increase can be sustainable if the environmental and health costs are also increased (Ma & Joachim, 2006).

Recently, organic management (OM) of cropland or orchard has received growing attention from researchers and has fascinated ordinary consumers (Ma & Sauerborn, 2006). OM is a more eco-friendly model of agriculture than the conventional management (CM), as OM prohibits the use of synthetic fertilizers, pesticides, herbicides, growth regulators and genetic engineering (Acs, Berentsen & Huirne, 2005; Luttikholt, 2007). OM focuses on the “wholeness” which systematically co-ordinates each links of the system (Scofield, 1986). I.e., uses ecological approaches comprehensively to control pests, weeds and manage the whole system (Verhoog et al., 2007). In comparison with CM, OM is featuring an efficient utilization of resources such as energy and by the enhancement of biodiversity (Astier et al., 2014; Fuller et al., 2005; Hole et al., 2005; Mäder et al., 2002). Therefore, OM is considered as an ideal combination of economic benefits, environmental sustainability (Azadi et al., 2011) and human health. However, as such ideal models are seldom realized, policy-makers are hesitant to support OM in countries that suffering from food shortage such as China.

Fruit orchards have been recognized as special “croplands” severely afflicted by synthetic fertilizers and pesticides (Nie et al., 2005; Yu et al., 2012). To ensure the safety and quality of fruits, organic fruit production has become an inevitable trend for the sustainable development of fruit industry (Granatstein, Kirby & Willer, 2013). In 2006, the world’s cultivated area of organic apples was 30,000 ha, and China’s acreage of organic apple was 1,580 ha (Zhang et al., 2009). Many researchers have carefully analyzed the differences between CM and OM orchards, and found that OM increased soil biological properties (Glover, Reganold & Andrews, 2000), enzyme activities (Floch, Capowiez & Criquet, 2009) and microbial biomass (Araújo, Santos & Monteiro, 2008). Although OM had lower yields than the CM, the former had crucial advantages against the latter, such as better fruit quality and popularity, higher profits and energy efficiency as well as less negative environmental impacts (Amarante et al., 2008; Reganold et al., 2001). We used cattle manure to improve soil quality and enlarge soil biota, applied ecological pest management (EPM) (Tshernyshev, 1995) to control pests, propagated the native species (*Duchesnea indica*) as groundcover to suppress pernicious weeds, and kept *Osmia excavata* for pollination. During such a new management process, all artificial synthetic chemicals were banned. The aim of this study was to investigate the effectiveness of integrating biodiversity management into the ecological process for the realization of sustainable orchard, by comparing both the ecological and economic effects between OM and CM of the apple orchard. We hypothesized that biodiversity management of an organic orchard based on the ecosystem balance and mutual restrictions among the species could be beneficial for soil improvement, soil biota richness, pests and weed control as well as eco-economic combined development.

## Methods

### Study site

The experiment was conducted in Hongyi Organic Farm, Pingyi County, Shandong Province, China (35°26′21″N, 117°50′11″E). The climate of the study area is characterized as typical temperate and monsoonal. The mean annual precipitation is 770.2 mm, with the rainfall concentrated in July (259 mm) and August (192 mm), and least precipitation in January (11 mm). The average annual temperature is 13.2 °C, with the maximum being in July (31 °C), and the minimum in January (–5 °C). The type of the soil is brown earth. The chemical and physical properties of 0–20 cm soil of OM and CM as well as cattle manure applied for the experiment are listed in Table 1.

**Table 1:** Initial chemical and physical properties of soil (0–20 cm layer) in organic management (OM) and conventional management (CM) and nutrients of cattle manure (on dry weight basis).

	Organic matter (g kg^−1^)	Total nitrogen (g kg^−1^)	Available phosphorus (mg kg^−1^)	Available potassium (mg kg^−1^)	pH	Water content (%)	Bulk density (g cm^−3^)
CM soil	17.8 ± 2.1	1.1 ± 0.1	146.3 ± 5.7	386.9 ± 16.7	6.4 ± 0.1	18.1 ± 0.02	1.3 ± 0.1
OM soil	18.5 ± 3.0	1.3 ± 0.1	135.4 ± 9.0	394.6 ± 7.6	6.3 ± 0.1	18.0 ± 0.02	1.4 ± 0.1
Cattle manure	420.5 ± 10.3	20.2 ± 0.8	366.5 ± 43.5	207.5 ± 42.5	7.7 ± 0.5	66.2 ± 3.8	–

**Notes.**

Data were measured in 2012, prior to implementation of treatments (means ± standard deviation, *n* = 3).

### Experimental design

Two adjoining apple orchards that located next to each other (separated by a 5 m production road) in the same field with different managements, organic management (OM) and conventional management (CM), were applied for the experiment. Each management had 3,000 m^2^ and was divided into three plots with an area of 1,000 m^2^. Soil type and climate were the same in OM and CM, with soil properties being quite similar. The cultivar of apples was Fuji and originated from Japan. All the trees were planted in 1998, at 3 m × 3 m distances. Soil and fruit sampling as well as earthworm, weed and pest investigations were done in the three plots as three replicates.

**Table 2:** Managements of organic management (OM) and conventional management (CM) on fertilization, sterilization, pollination, pest control and weed control (2012–2014).

Treatments	CM		OM	
**Fertilization**	Compound fertilizer of potassium sulfate: 5, 310 kg ha^−1^		Cattle manure: 217.5t ha^−1^	
	Urea: 1,500 kg ha^−1^	
**Pest control**	Chemical pesticides	Mar. Imidacloprid, Beta-cypermethrin; 1 time	Biological methods	Mar. Lime sulfur 1 time
		May. Chlorpyrifos, Hexythiazox; each 1 time		May. Biogas slurry 1 time
		Jun. Azacyclotin, Imidacloprid, Chlorbenzuron; each 1 time		Jun. Biogas slurry 1 time
				Natural enemies
		Jul., Aug. & Sep. Chlorbenzuron, Hexythiazox and Beta-cypermethrin; once a month	Physical methods	Frequency trembler lamps
				Worm sticky traps
				Stem residue traps
**Sterilization**	Pesticides	Mar. Mancozeb, Thiophanate methyl, each 1 time	May Bordeaux mixture, 1 time	
		May Propineb, tebuconazole, Mannitol chelating calcium, Carbendazim, Mancozeb, each 1 time	Jul. Bordeaux mixture, 1 time	
			Aug. Bordeaux mixture, 1 time	
		Jul. Carbendazim		
		Aug. Mancozeb		
		Sep. Carbendazim		
**Weed management**	Herbicides (paraquat): 3 times		Biological: Mar. 2012 propagating * D. indica* (spaced 1 × 1 m)	
	Physical: mowing in Mar. 1 time	
**Pollination**	Artificial supplementary pollination		Keeping insect pollinators: *O. excavata*	

Details of managements for each year are shown in Table 2. The CM was treated with synthetic fertilizers, which including urea (N = 46%) and compound fertilizer of potassium sulfate (N = 15%, *P*_2_*O*_5_ = 15%, *K*_2_*O* = 15%). The OM was fertilized with cattle manure (nutrient content is shown in Table 1). The total amount of fertilizer input in the two systems was set according to the average application rate of fertilizers in the main apple production area of Shandong Peninsula; the organic manure is slow-release fertilizer and for the purpose of compensating the nutrient loss caused by the growing grass were also taken into account. The total amount of nitrogen inputs in the two systems were controlled equivalent at the dose of 1,494 kg ha^−1^. Non-selective pesticides, herbicides and fungicides were used in the CM just as most local orchards did and these agrochemicals contained different toxic agents such as beta-cypermethrin, parathion (Nie et al., 2005). The dose rate of the pesticides and herbicides was in accord with the instructions of the chemical products. Instead, in OM, no synthetic chemicals were applied and the EPM method was adopted to control pest; the bordeaux mixture was applied to prevent disease (Vega, Escobar & Velázquez-Martí, 2013). One native plant species (*Duchesnea indica*) was propagated in the spring of 2012 for suppressing harmful weeds growth, through planting branches and mowing other weeds simultaneously. Domesticated bees (*Osmia excavata*) were kept for pollination in OM. This experiment continued for three years (2012–2014).

### Soil sampling and analysis

Five subsamples were randomly taken in each plot at 0–20 cm layer using a soil auger of 5 cm in diameter and mixed together into one soil sample. Hence, each management had three replications. The soil samples were divided into two parts. One part was air-dried and passed through a 100 mesh sieve for analysis of soil physicochemical properties, and the other part was stored at –20 °C for microbiological analysis.

Soil total nitrogen was determined by the Kjeldahl method (Bremner & Mulvaney, 1982). Soil organic carbon was measured by potassium dichromate oxidation-ferrous sulphate titrimetry method (Nelson & Sommers, 1996). Soil microbial biomass C and microbial biomass N were analyzed following the procedure of chloroform fumigation extraction (Brookes et al., 1985; Wu et al., 1990).

### Amplification of 16s rDNA and sequencing analysis of bacterial communities

Soil samples collected on Jun 21st, Dec 22nd, 2013 and Jun 10th, Dec 8th, 2014 were applied for molecular analysis. The sample codes with “S” stood for summer and “W” for winter. DNA was extracted from 1 g soil samples using CTAB (Hexadecyltrimethy Ammonium Bromide) method (Konstantinos et al., 2011). Bacterial 16S rDNA at V4 hypervariable region was amplified using specific primer set 515F (5′-GTGCCAGCMGCCGCGGTAA-3′) and 806R (5′-GGACTACHVGGGTWTCTAAT-3′) (Bates et al., 2010). Each primer contained 8–13 bp paired-end error-correcting barcodes (Table S1) (Fadrosh et al., 2014). All PCR reactions were carried out with Phusion^®^ High-Fidelity PCR Master Mix (New England Biolabs) and PCR products were purified with Qiagen Gel Extraction Kit (Qiagen, Hilden, Germany).

Sequencing libraries were generated using TruSeq^®^ DNA PCR-Free Sample Preparation Kit (Illumina, San Diego, CA, USA) following manufacturer’s recommendations and index codes were added. The library was sequenced on an Illumina MiSeq platform and 300 bp paired-end reads were generated and then merged using FLASH (Magoc & Salzberg, 2011). Quality filtering on the raw tags were performed to obtain the high-quality clean tags according to the QIIME (Caporaso et al., 2010) and UCHIME algorithm (Edgar et al., 2011) was used to remove the chimera sequences in order to obtain the effective tags. Sequences with ≥97% similarity were assigned to the same OTUs using UPARSE software (Edgar, 2013). Representative sequence for each OTU was screened for further taxonomic annotation with the GreenGene Database (DeSantis et al., 2006) based on RDP classifier (Wang et al., 2007). The estimated species richness was demonstrated with rarefaction curves, Chao 1, Shannon’s index and relative abundance.

### Earthworm abundance evaluation

Earthworms in the soil were qualified through hand-sorting method trimonthly in each experimental year. Three soil blocks were investigated in each plot at one sampling time. The size of the soil blocks was 30 cm (length) × 30 cm (width) × 20 cm (depth). The earthworms collected were brought to the laboratory to be identified and counted.

### Weed biodiversity assessment

Weed communities were assessed in mid-July of each year. Three quadrats of 1 m × 1 m size within inter-row area were randomly identified in each plot of OM and CM, assessed the number and coverage of each species. Biodiversity indices including Simpson index (*D*), Shannon–Wiener diversity index (*H*′), and Pielou index (Hill, 1973; Pielou, 1969) were calculated by the following Eqs. (1)–(3):

(1) \begin{eqnarray*}D=1-\sum _{i=1}^{s}({N}_{i}/N)^{2}\end{eqnarray*}

(2) \begin{eqnarray*}{H}^{^{\prime}}=-\sum _{i=1}^{s}Pi\hspace*{1em}\ln Pi\end{eqnarray*}

(3) \begin{eqnarray*}E={H}^{^{\prime}}/\ln S.\end{eqnarray*}

### Monitoring of phototactic pest dynamics

Three frequency trembler lamps with short wave (330 nm) were installed equidistantly in the three plots of OM to capture the phototactic pests. The light-traps had photosensitive automatic switch which turned the light on at night and off in the daytime. We collected the captured pests every morning from May to October in 2012 to 2014. The captured pests were categorized as scarab beetles, moths, and other small insects and then weighed separately.

### Apple yields assessment

Measurements of yields were conducted at harvest time in each year. Ten trees were randomly selected in each plot of OM and CM, weighed all of the fruits and we calculated the apple yield per plant. The yields of both OM and CM were obtained by average yield per plant multiplying planting density.

### Pesticide residue determination of fruits

Although none chemicals were used in the OM, organic apples were also tested by the same multi-residue analysis as CM. A total of 191 kinds of pesticides and herbicides categories were assayed by gas chromatography or liquid chromatography according to different chemical components (Table S2). The method and examination standard for testing pesticide residue were based on China national food safety standard-Maximum residue limits for pesticides in food (GB2763-2014).

### Economic benefits assessing

The total inputs and outputs of two apple managements were recorded in details, with the output–input ratio being calculated individually. The apple price was fixed according to the different quality grades. The CM apples were all sold in the local market, while part of the OM ones with good appearance were sold through online shop and bulk orders at high price. Meanwhile, a part of OM apples were sold at low price due to the small fruit weight. The packing charges as well as the labor cost of packing and selling were deducted.

### Statistical analysis

The annual mean values used in the figures were calculated from the quarterly mean values. All data were analyzed using one-way analysis of variance (ANOVA), and the differences between CM and OM were tested by two-sample Student’s *t*-test and the differences between experimental years were tested by * LSD* (least significant difference) test with SPSS 16.0. Differences between managements were identified as significant if *P* < 0.05 and highly significant if *P* < 0.01. Figures were performed using Sigma Plot 10.0 (Aspire Software Intl. Ashburn, VA, USA). All the data of the results were means ± standard error.

## Results

### Improvement of soil characteristics

As is shown in Table 3, from 2012 to 2014, soil organic carbon content (SOC) of 0–20 cm layer in OM remarkably increased, from 15.6 g kg^−1^ to 22.7 g kg^−1^. SOC of CM increased in the first year of survey but decreased from 2013 to 2014, which might be caused by longtime application of synthetic fertilizers as well as herbicides, resulting in soil fertility degradation and instability. The differences of soil features between the two different systems were striking in the first two years of the experiment, and highly significant in the third year (*P* < 0.01). For instance, SOC of OM was 2.1 times higher than that of CM in 2014 (Table 3). Total nitrogen (TN) content of OM was higher than that of CM and the difference was obvious (*P* < 0.05) in 2014 by a factor of 1.97. However, it was not statistically significant in 2012 and 2013 (*P* > 0.05). The same as SOC, TN of CM also elevated at the beginning then decreased later on, which might be caused by nitrogen loss of ammonia volatilization and nitrate nitrogen leaching under low C/N ratio condition.

**Table 3:** SOC, TN, MBC and MBN of 0–20 cm soil layer in organic management (OM) and conventional management (CM) during 2012–2014.

		CM	OM
SOC (g kg^−1^)	2012	8.54 ± 1.96Bb	15.59 ± 1.89Ab
	2013	15.31 ± 1.50Ba	21.29 ± 1.29Aa
	2014	11.08 ± 0.55Bab	22.73 ± 1.82Aa
TN (g kg^−1^)	2012	1.06 ± 0.19Ab	1.12 ± 0.14Ab
	2013	1.53 ± 0.14Aa	1.90 ± 0.16Aab
	2014	1.26 ± 0.08Bab	2.47 ± 0.38Aa
C/N ratio	2012	7.90 ± 0.66Ab	9.83 ± 0.58Aa
	2013	10.00 ± 0.32Aa	11.36 ± 0.87Aa
	2014	8.85 ± 0.15Aab	9.50 ± 0.67Aa
MBC (mg kg^−1^)	2013	134.05 ± 20.14Aa	220.30 ± 28.71Ab
	2014	164.47 ± 28.01Ba	376.13 ± 35.38Aa
MBN (mg kg^−1^)	2013	6.32 ± 2.40Aa	15.47 ± 2.03Ab
	2014	10.08 ± 4.82Ba	31.39 ± 5.18Aa

**Notes.**

Data are means ± standard error. Bars with different capital letters indicate significant difference at *P* < 0.05 level (Student’s *T*-test) between two managements, and bars with different lowercase letters indicate significant difference at *P* < 0.05 (ANOVA, *LSD* test) among sampling years.

As is shown in Table 3, microbial biomass carbon (MBC) of OM increased substantially from 220 mg kg^−1^ to 376 mg kg^−1^, which was significantly higher than that of CM in 2014 (*P* < 0.05). MBC of CM was 134 mg kg^−1^ and 164 mg kg^−1^, respectively in 2013 and 2014. Microbial biomass nitrogen (MBN) also displayed an obvious growth in OM, increased by 102% from 2013 to 2014. MBN from CM kept at a markedly lower level, being 30% of that of OM in 2014 (*P* < 0.05).

### Soil bacterial community

**Figure 1:**
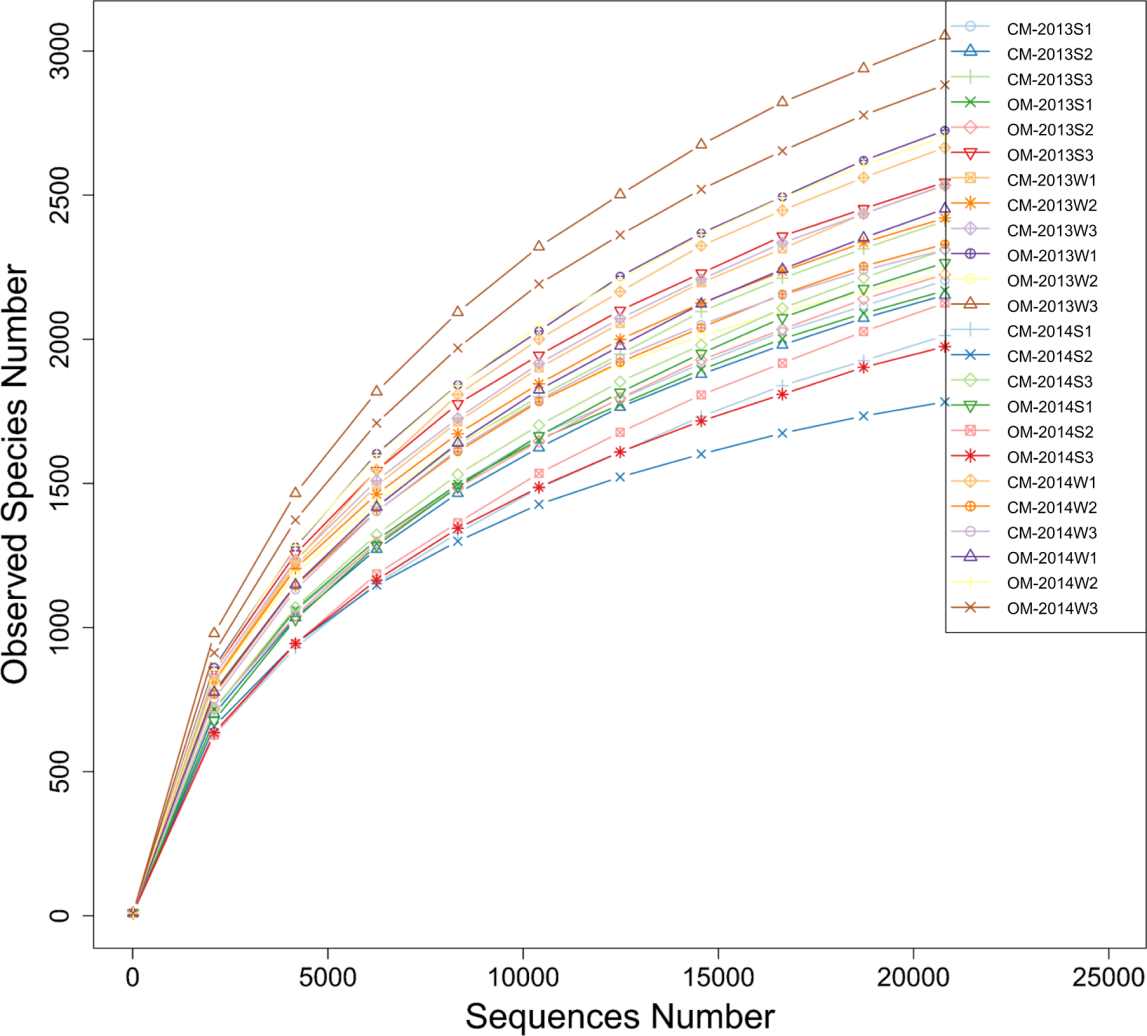
Rarefaction curves of observed species (i.e., OTUs) number clustered at the 3% phylogenetic distance level based on the 16S rDNA gene sequences of all soil samples derived from organic management (OM) and conventional management (CM). “S” and “W” in the sample codes mean sampling time of summer at mid-June and winter at mid-December, respectively. Each management had three replicates.

**Figure 2:**
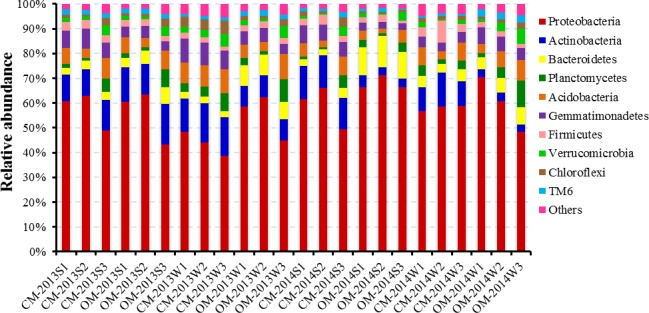
Relative abundance of the dominant bacterial at phylum level of soil samples derived from organic management (OM) and conventional management (CM) at summer and winter in 2013 and 2014 respectively. “S” and “W” in the sample codes mean sampling time of summer at mid-June and winter at mid-December, respectively. Each management had three replicates.

A total of 1,137,310 PE reads were obtained from the 24 soil samples derived from CM and OM in both summer and winter time of 2013 and 2014. At the 3% phylogenetic distance level, most rarefaction curves reached saturation, indicating that the majority of the OTUs had been covered (Fig. 1). As is shown in Fig. 2, the dominant bacterial phyla of the total soil samples were *Proteobacteria* (57%) and *Actinobacteria* (10%), with others including *Acidobacteria* (6%), *Gemmatimonadetes* (6%), *Bacteroidetes* (5%), *Planctomycetes* (4%), *Firmicutes* (3%), *Verrucomicrobia* (2%), *Chloroflexi* (2%), *TM6*(2%) and so on. Soil bacterial abundance of CM was significantly richer than that of OM in *Actinobacteria*(except summer 2013), *Gemmatimonadetes* (except winter 2013) and *Chloroflexi* (winter 2013) at phyla level (*P* < 0.05). By contrast, OM samples were evidently more abundant in *Bacteroidetes* (winter 2013, summer 2014), *Gemmatimonadetes* (winter 2014), *TM6* (winter of both year). However, none significant differences (*P* > 0.05) were found at other sampling times (Fig. 2).

**Figure 3:**
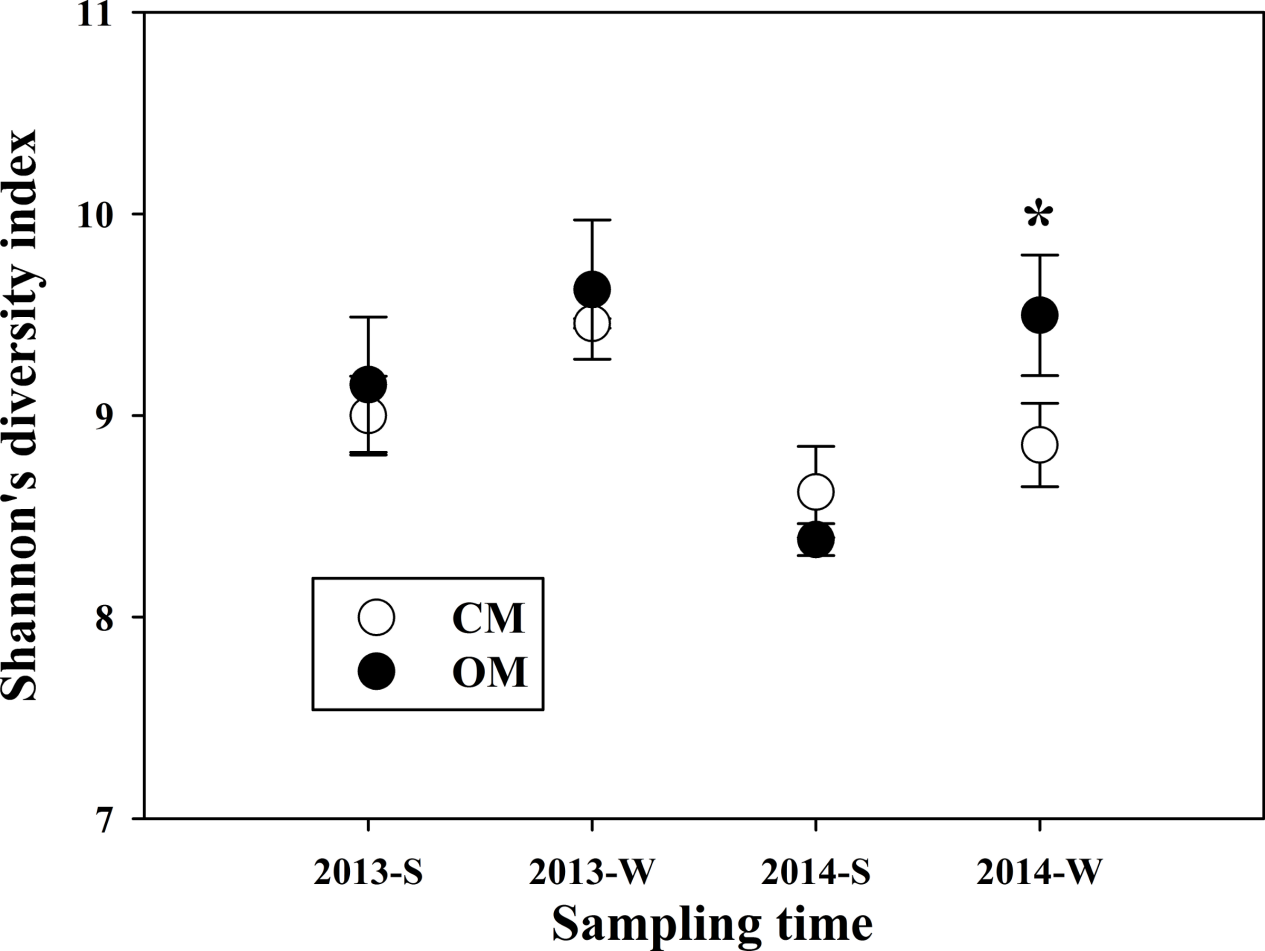
Shannon’s diversity index of soil bacteria community of organic management (OM) and conventional management (CM) in summer and winter of 2013 and 2014 respectively. Data are means ± standard error. “S” and “W” in the sample codes mean summer at mid-June and winter at mid-December respectively. * indicates the significant difference between managements at *P* < 0.05 level (Student’s *t*-test).

Despite richer relative abundance for several phyla, CM soils were found to be poorer in bacteria community as a whole. Except at the winter of 2013, Shannon’s diversity index of CM was lower than that of OM at the other three sampling times, notably in winter 2014 with a gap of 7% (Fig. 3). The detected rhizobia of the soil samples included *Devosia*, *Burkholderia*, *Rhizobium*, *Cupriavidus*, *Mesorhizobium* and *Ochrobactrum* (Fig. S1). The sum of these six genera’s relative abundances was a little bit higher in OM than that in CM at the four sampling periods separately, however the differences were not prominent (*P* > 0.05). Besides, the relative abundance of some other functional bacteria genus such as *Pseudomonas*, *Flavobacterium*, *Gemmata*, are higher in OM than those in the CM.

### Earthworm abundance

**Figure 4:**
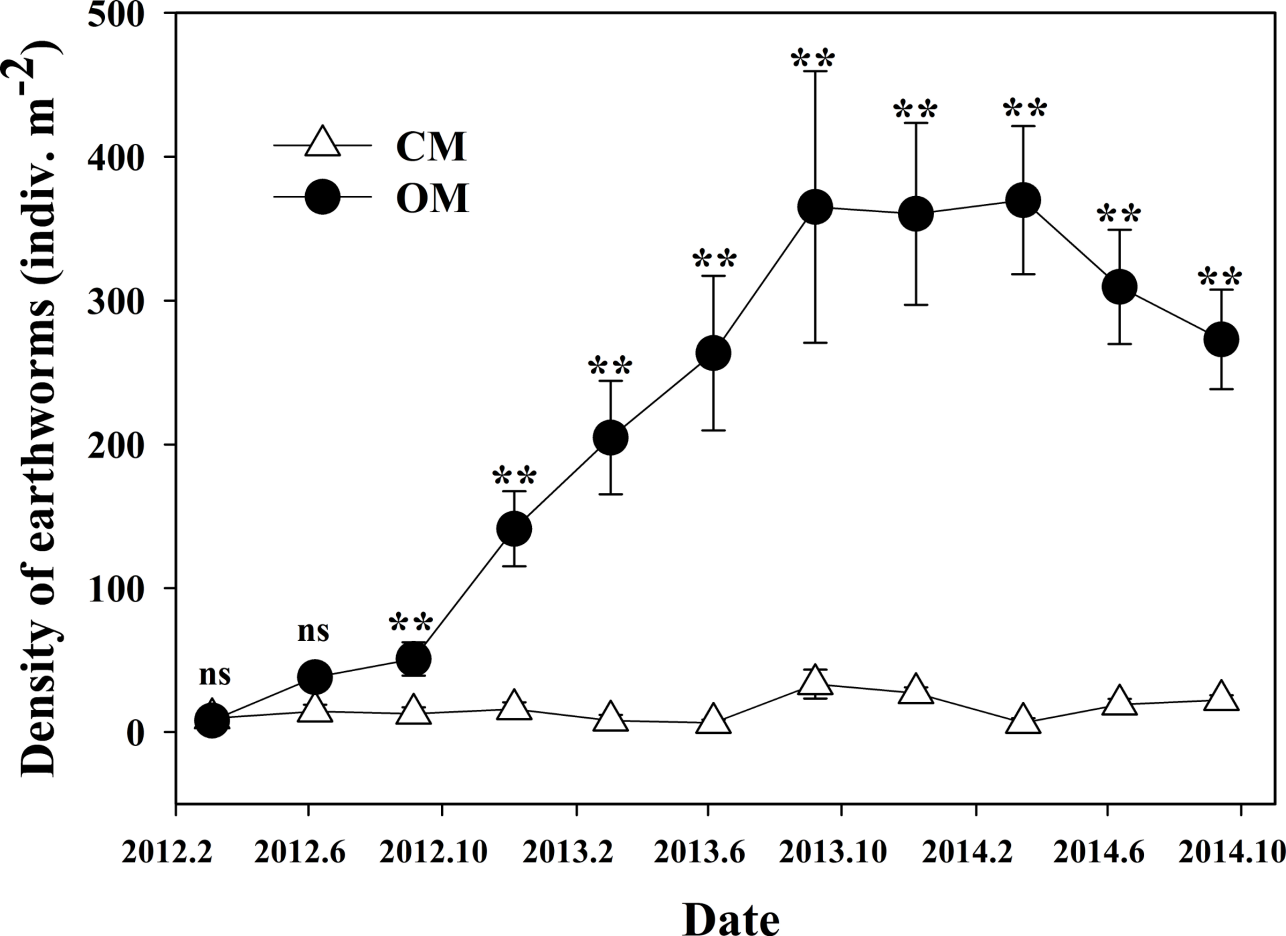
Dynamics of earthworm density under organic management (OM) and conventional management (CM) during 2012∾2014. Data are means ± standard error. ** indicates significant difference between two managements within each sampling time at *P* < 0.01 level and “ns” means non-significant difference at *P* < 0.05 level (Student’s *t*-test).

The density of earthworms in OM was consecutively increased in the first two years, from 8 m^−2^ to 365 m^−2^ and peaked at the level of 369 m^−2^ at the beginning of the third experimental year. However, it declined to 273 m^−2^ later (Fig. 4), which may be owing to the density feedback under limited carrying capacity. By contract, the CM held fewer earthworms around 6∾33 m^−2^, which remained at an exceptionally lower level (*P* < 0.01). In 2014, OM had an average of 20 times more earthworms than CM, and the largest gap of density was more than 50 times appeared in the spring of 2014 (Fig. 4).

Through identification of samples, we found that earthworms in the two managements mainly included *Amynthas heterochaetus* and *Drawida japonica*. Earthworms were classified into detritivores and geophages (Lee, 1985) based on whether feeding on rich humus or not, reflecting characteristics of composition under the two managements. It was obvious that due to the rich organic matter input, the density of detritivores in OM was extremely higher than that of CM (*P* < 0.01) (Fig. S2A), while the difference of geophages’ density was quite smaller between the two managements (*P* < 0.01) (Fig. S2B).

### Weed biodiversity

**Figure 5:**
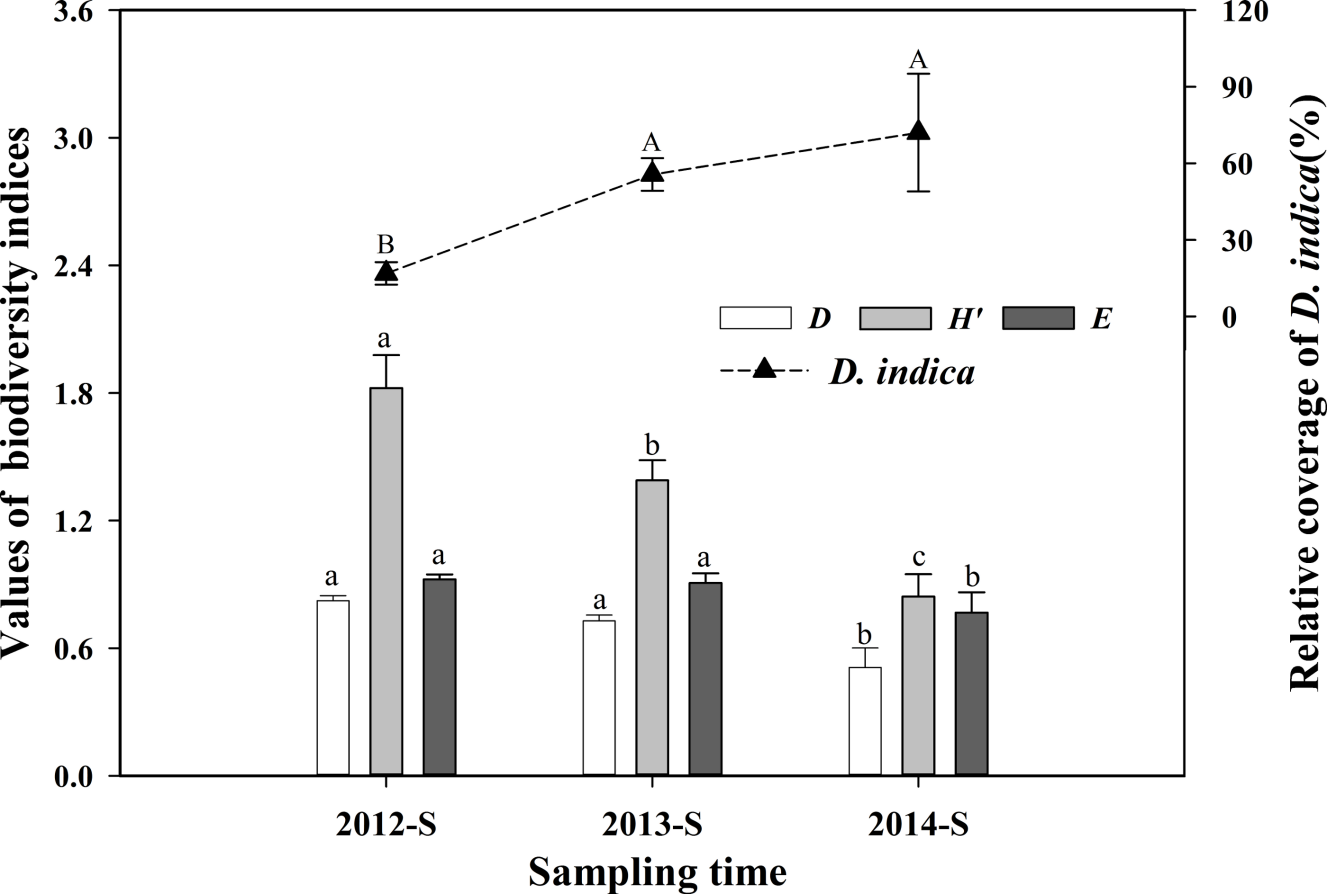
The *D. indica’*s relative coverage and its propagating influences on the biodiversity indices of weed communities at each summer from 2012 to 2014 in OM. *D*, Simpson index; *H*′, Shannon–Wiener index; *E*, Pielou index. “S” for sampling time means summer at mid-July. Data are means ± standard error. Different capital letters indicate significant difference of the relative coverage of *D. indica* and different lowercase letters indicate significant difference of each biodiversity index at *P* < 0.05 level among sampling years separately (ANOVA, *LSD* test).

In CM, weeds were extirpated by chemical herbicides. In OM, however, due to the strong clonal propagation capacity, *D. indica* grew faster than harmful weeds in early spring, so it became obviously dominate. The relative cover of *D. indica* increased substantially by 3.3 folds from 2012 to 2014 and reached 72% (Fig. 5). Simpson index (*D*), Shannon–Wiener index (*H*′), and Pielou index (*E*) of weed communities showed the same decreasing trend, reducing by 38%, 54% and 17% respectively from 2012 to 2014 (Fig. 5). Nevertheless, declines of Simpson index and Pielou index were not remarkable between the first two years but noteworthy between the last two years. Reduction of Shannon–Wiener index was significant among the three years (Fig. 5).

### Dynamics of nocturnal phototactic pests

**Figure 6:**
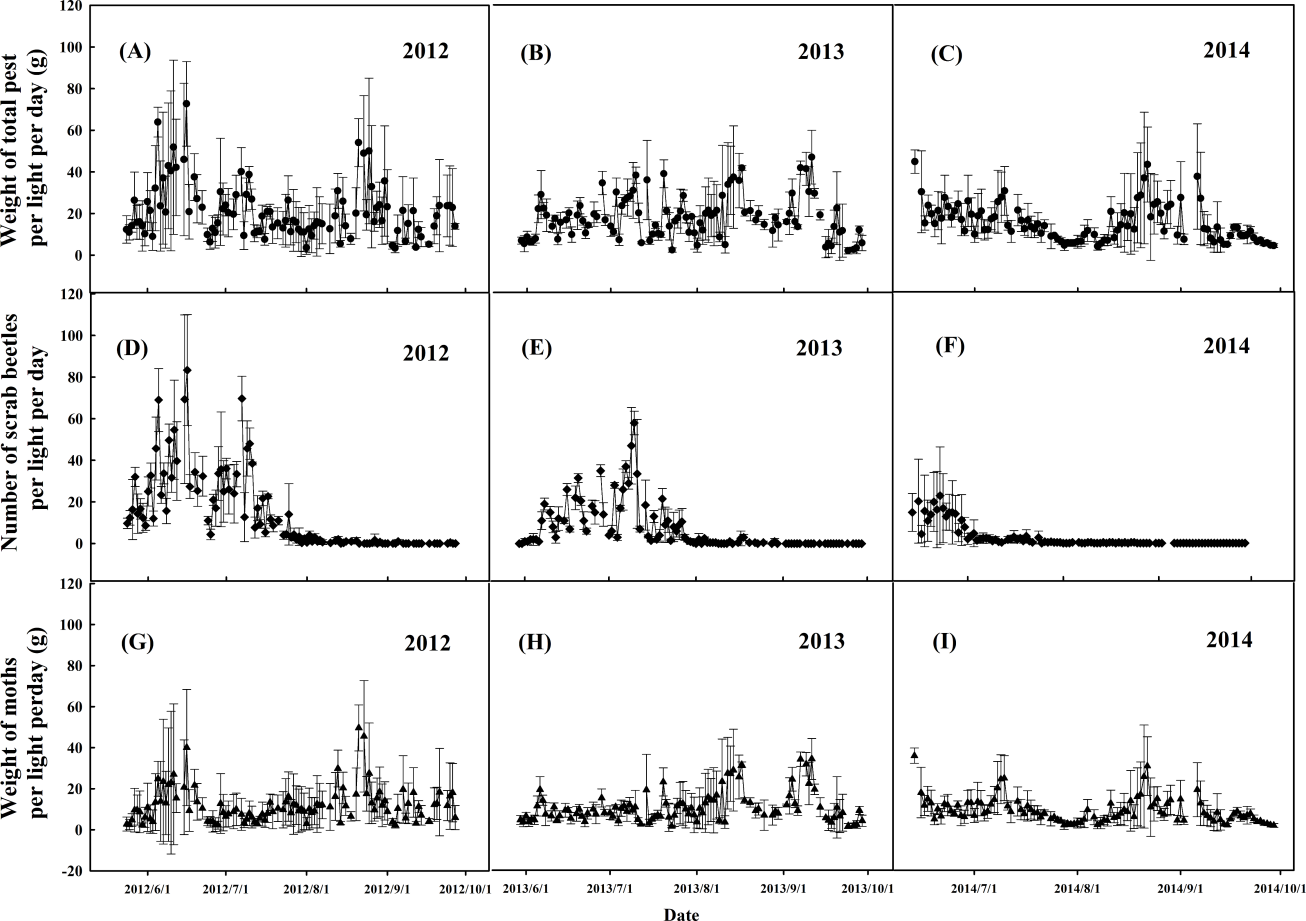
Dynamics of phototatic pests captured by the frequency trembler lamps monitored in OM during 2012–2014. (A–C), Weight of total phototatic pests; (D–E), Population dynamics of scarab beetles; (G–I), Weight of moths. Data are means ± standard error.

In CM, pests were controlled by chemical broad spectrum pesticides. In OM, continual trapping and monitoring of nocturnal phototactic pests was carried out for the whole growth seasons of 2012–2014. The captured phototactic pests mainly included Lepidoptera, Coleopteran, Orthoptera, and Hemiptera. The amount of total daily captured pests displayed a generally declining tendency, so did the scarab beetles and moths (Fig. 6). According to our monitoring results, the average weight of total daily captured pests per light dropped from 21 g day^−1^ to 13.7 g day^−1^ by 35% reduction from 2012 to 2014 (Figs. 6A–6C). The average number of daily captured scarab beetles had greatly shrunk from 14 day^−1^ to 2 day^−1^, or by 86% decrease (Figs. 6D–6F). The peak number of scarab beetles population at outbreak has decreased sharply from 83 day^−1^ to 23 day^−1^, by 72% decrease, against the time node of zero-value being shifted forward from August to July (Figs. 6D–6F). The trapped moths involved many species, which mainly had two or three activity peaks in summer and the beginning of autumn. The average weight of daily captured moths declined from 11.9 g day^−1^ to 9.2 g day^−1^, with a reduction of 23% (Figs. 6G–6I).

### Yields and economic benefits

**Figure 7:**
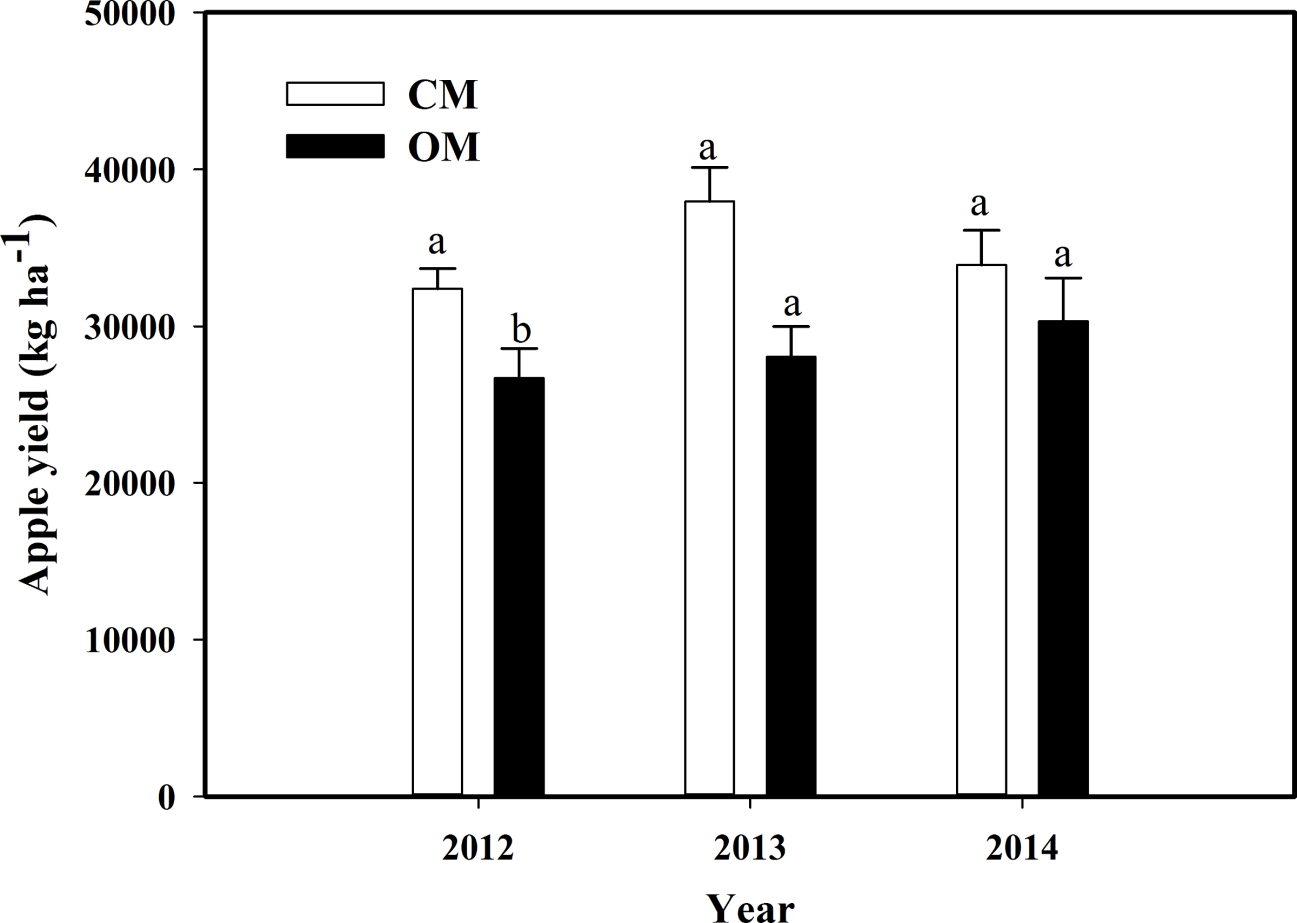
The yields of organic management (OM) and conventional management (CM) during 2012–2014. Data are means ± standard error. Bars with different lowercase letters indicate significant difference at *P* < 0.05 level (Student’s *T*-test) between two managements in the same year.

The apple yield of OM was lower than that of CM in each of the three years of the experiment, by 16.7%, 20.2%, and 12.2% respectively (Fig. 7). The difference of yield between the managements was significant in 2012 (*P* < 0.05) and not notable in the last two years (*P* > 0.05). The yield of the OM had a slow but steady rising tendency, while the yield of CM showed a downward trend after the rise in the second year.

**Table 4:** Analysis on inputs and outputs of organic management (OM) and conventional management (CM).

	Details	CM ($ ha^−1^)	OM ($ ha^−1^)
Input	**Materials and equipment**		
	Fertilizer	3,152	1,053
	Pest & disease control	1,033	1,463
	Weed control	73	
	Depreciation of equipment	90	160
	Other materials (apple bags, gasoline, etc.)	127	654
	**Labor**		
	Fertilization	436	1,598
	Pest & disease control	2,324	1,307
	Weed control	109	363
	Irrigation	145	145
	Pollination	291	0
	Other daily management	3,922	3,269
	Irrigation (water cost)	872	872
	Electricity spraying and irrigation	129	67
	Transportation for manure and biogas slurry	0	358
Annual average input		12,703	11,309
Output	2012	21,788	37,536
	2013	24,233	42,345
	2014	22,953	44,682
Annual average output		22,991	41,521
Output/input ratio	Annual average output/Annual average input	1.81	3.67

**Notes.**

Analysis are based on actual sales data and calculation details are shown in Table S3.

As is shown in Table 4 (see Table S3 for calculating details of input and output), CM invested mainly on synthetic fertilizers and pesticides, and cost more in terms of labors due to the high pesticides spray frequency and artificial pollination. Although OM was somewhat labor-intensive, especially in cattle manure fertilization and weed control, it saved some labor because of physical pest controlling and natural honeybee (*O. excavata*) pollination. In addition, OM cost more in purchasing equipment and setting paper bags. Therefore, the total inputs of two systems were approximately the same.

As for food security, a detection report for 191 items of chemical pesticides and herbicides residue (Table S2) indicated that organic apples can meet EU’s organic food standards with none pesticides residue found. Residues of pesticides including chlorbenzuron, chlorpyrifos and fungicide namely tebuconazole were found in conventional apples. Accordingly, as shown in Table S3, the organic apples possessed higher marketable price with an average price of $2.03 kg^−1^ and were mainly sold to high-consuming customers in large cities such as Beijing, Shanghai and Guangzhou, resulting in relatively higher economic outputs. In contrast, the conventional apples were sold in the local market at a lower price with an average of $0.56 kg^−1^. In consequence, OM displayed much higher economic benefits, with the output–input ratio being 3.67 or 103% higher than that of CM.

## Discussion

It is well documented that the extensive use of synthetic chemicals in agroecosystem had adverse impacts on bacterial diversity and activity (Johnsen et al., 2001). We here reported that high amount of application of organic fertilizer containing plenty of organic matter contributed to increase soil organic carbon (SOC), and further promoted soil microorganism’s activity, resulting in the increase of microbial biomass carbon. High input of organic fertilizer could improve soil quality rapidly. We also discovered that under the pollution-free soil environment such as in the organic management (OM) method of apple orchard, microorganisms were capable to multiply massively with higher biodiversity. Such a result was in line with previous researches (Araújo, Santos & Monteiro, 2008; Araújo et al., 2009). We adopted 16S rDNA high-throughput sequencing based on the Illumina MiSeq (PE300) platform to evaluate bacterial community accurately. The higher Shannon’s index of bacteria in OM at the later stage of the experiment indicated richer soil bacteria which was induced by the gradually increasing SOC. Correspondingly, abundant bacteria promoted biogeochemical cycle, which played a critical role in the humus-formation. Some researchers also found that OM had high richness of arbuscular mycorrhizal fungi (Purin, Filho & Stürmer, 2006). It is worth to mention that the total relative abundance of rhizobia, which can fix nitrogen directly from the air, was slightly larger in OM than in the conventional management (CM) model, displaying a growth trend. The abundance of rhizobia is simply driven by N availability in the soil. The results indicated a lower availability of N in OM and it secured the system from uncontrolled N-losses.

Earthworm is an important group of soil fauna that participates in the nutrient cycle and modifies soil physicochemical properties through burrowing, foraging and excreting. Our research confirmed that organic fertilizer has distinctly positive effects on earthworms’ growth and reproduction, in agreement with previous researches (Domínguez et al., 2014; Lapied, Nahmani & Rousseau, 2009). More distinctly, earthworms in OM showed much higher increasing density, and the dominant group was detritivores preferring rich humus. This result might be attributed to the years of ever-rising organic matter inputs as well as none application of toxic herbicides and pesticides (Reinecke & Reinecke, 2007; Zaller et al., 2014). Higher density of earthworms indicated healthy soil environment, since earthworms could increase water penetration, help roots extension, and improve soil fertility by worm cast. Additionally, earthworms can help boost plant production as well (Van Groenigen et al., 2014).

*D. indica* is a native perennial stoloniferous herbaceous plant that reproduces rapidly at a high ramet density through clonal growth and sympodial branching configurations (Dong, Zhang & Chen, 2000). Although there is niche overlap (Colwell & Futuyma, 1971) between* D. indica* and noxious weeds, the phenological period of* D. indica*’s is earlier than that of some weeds and *D. indica* can win the niche competition. Increasing relative cover of *D. indica* as well as declining biodiversity indices of weeds indicated that *D. indica* expanded rapidly and successfully supressed harmful weeds, turning into the dominant species of the understory herbaceous layers. Therefore, we found *D. indica* is an ideal native ground-cover plant for controlling weeds based on the ecological principles, and this method can remove three-time application of long residual herbicides.

In OM, much more harmonious relationships existed between ground cover and soil biota. Groundcover of *D. indica* is beneficial to improve the soil microenvironment by maintaining soil moisture as well as tempreture. The litter can be reincorporated into the soil as a supplement of organic matter and promoting microorganism’s activity (Wardle et al., 2001). The groundcover of *D. indica* can provide advantageous habitats for soil microfauna and natural enemies as well. Earthworms, especially detritivores, interact with microbes consumingly in the process of decomposition of organic matter, affecting the microbe community through an external approach, burrowing, foraging, excreting and through an internal approach by the earthworm gut (Brown, 1995; Domínguez, Aira & Gómez-Brandón, 2010). Aboveground and belowground biodiversity interact positively on reciprocal benefit together, resulting in the mutual developments. This would contribute to improve the soil’s biological characteristics and the apple trees’ growth (Chen et al., 2014; Shen et al., 2009).

Frequency trembler lamps are effective for capturing nocturnal phototactic pest, especially for Lepidoptera, Coleoptera and Orthoptera species. This method could control populations of adults to reduce spawning in the early stages of outbreaks, thus decreasing their offspings. Most of the natural enemies of pest were diurnal and seldom appear in the night, few having phototaxis characteriatics. Therefore, it is an environmentally friendly approach to control pests without harming diurnal helpful insects and natural enemies like birds and ladybugs. We have observed that in OM, the number of natural enemies and herbivories were extensively more than CM due to the absence of pesticides as well as herbicides. For instance, *Harmonia axyridis* and *Hyperaspis repensis* which prey abundantly on *Aphis ciricola* and *Myzus malisuctus*, showed rapid growth in population. Besides, *Henosepilachna vigintioctopunctata*, which is a plant-eater, also multiplied massively, and they feed on Solanum nigrum, which is a kind of common weed.

Based on our economic analysis, we found that few differences of total inputs exsited between the two systems. Even though the yield of OM was much lower than that of CM, organic apples were sold at premium marketable price by 4–5 folds higher than that of conventional apples at the same grade because of higher quality. Thus, the price advantage could make up for the yield losses. Apparently higher profits in OM have been achieved, which is also consistent with previous studies about apple orchard (Swezey et al., 2009) and kiwifruit orchard (Müller et al., 2015). It is of particular importance that OM achieved safety fruits and higher economic benefits through the totally healthy and pollution-free production. On account of consumers’ demand for safe food and farmers’s demand for increasing revenue, it is urgent for policy makers and farmers to convert conventional cultivation into organic cultivation with reasonable biodiversity managements.

## Conclusions

Biodiversity management in the organic apple orchard could obtain up to 103% higher economic benefits than chemical practices and maintain high ecological profits, on the premise of no irreversible damages to the environment and biodiversity. Integrated management of biodiversity in the organic system have been tested effectively for weed and pest control, soil biota abundance, soil quality improvement and to ensure food safety. The application of organic fertilizers enhanced the utilization of waste of the livestock farms. To meet the urgent demand for agroecosystem conservation and the consumers’ requirement for healthy fruits, organic orchard farming could be an alternative option for farmers in the shifting process from chemical to a sustainable organic practice.

##  Supplemental Information

10.7717/peerj.2137/supp-1 Figure S1 Relative abundance of the rhizobia at genus level of soil samples derived from organic management (OM) and conventional management (CM) at summer and winter in 2013 and 2014 respectively “S” for sampling time means summer at mid-June and “W” means winter at mid-December in each year.

10.7717/peerj.2137/supp-2 Figure S2 Earthworm community composition consisting of detritivores (A) and geophages (B) under organic management (OM) and conventional management (CM) Data are means + standard error. Bars with different capital letters mean significant difference at *P* < 0.01 level (Student’s *t*-test) between two treatments within each sampling time.

10.7717/peerj.2137/supp-3 Table S1 Barcode sequences used for respective soil samples of organic management (OM) and conventional management (CM)

10.7717/peerj.2137/supp-4 Table S2 Detection results of 191 kinds of chemical pesticides and herbicides residues in apple fruits of organic management (OM) and conventional management (CM) (Unit: mg kg^−1^) LC is short for Liquid chromatography; GC is short for Gas chromatography. All of the pesticides residues were less than the limit of detection which met the EU’s organic food standards.

10.7717/peerj.2137/supp-5 Table S3 Details of analysis on inputs and outputs of organic management (OM) and conventional management (CM) Apples were divided into six grades according to the marketable price, which was decided by the weight and appearance of the fruits. Total output was the sum of all the output in each class, which was calculated through yield of each class multiply unit price. Average annual output was the average of the output of the three years. Output/input ratio was calculate through dividing annual average output by annual average input.

## Additional Information and Declarations

### Data Availability

The following information was supplied regarding data availability: NCBI: Sequence Read Archive (SRA) database SRA: SRP067293
Bioproject: PRJNA305786.
