# Supplementary figures and images for: Biodiversity management of organic orchard enhances both ecological and economic profitability
Source: PeerJ. 2016 Jun 23;4:e2137. doi: 10.7717/peerj.2137 (PMC4924131; doi:10.7717/peerj.2137)

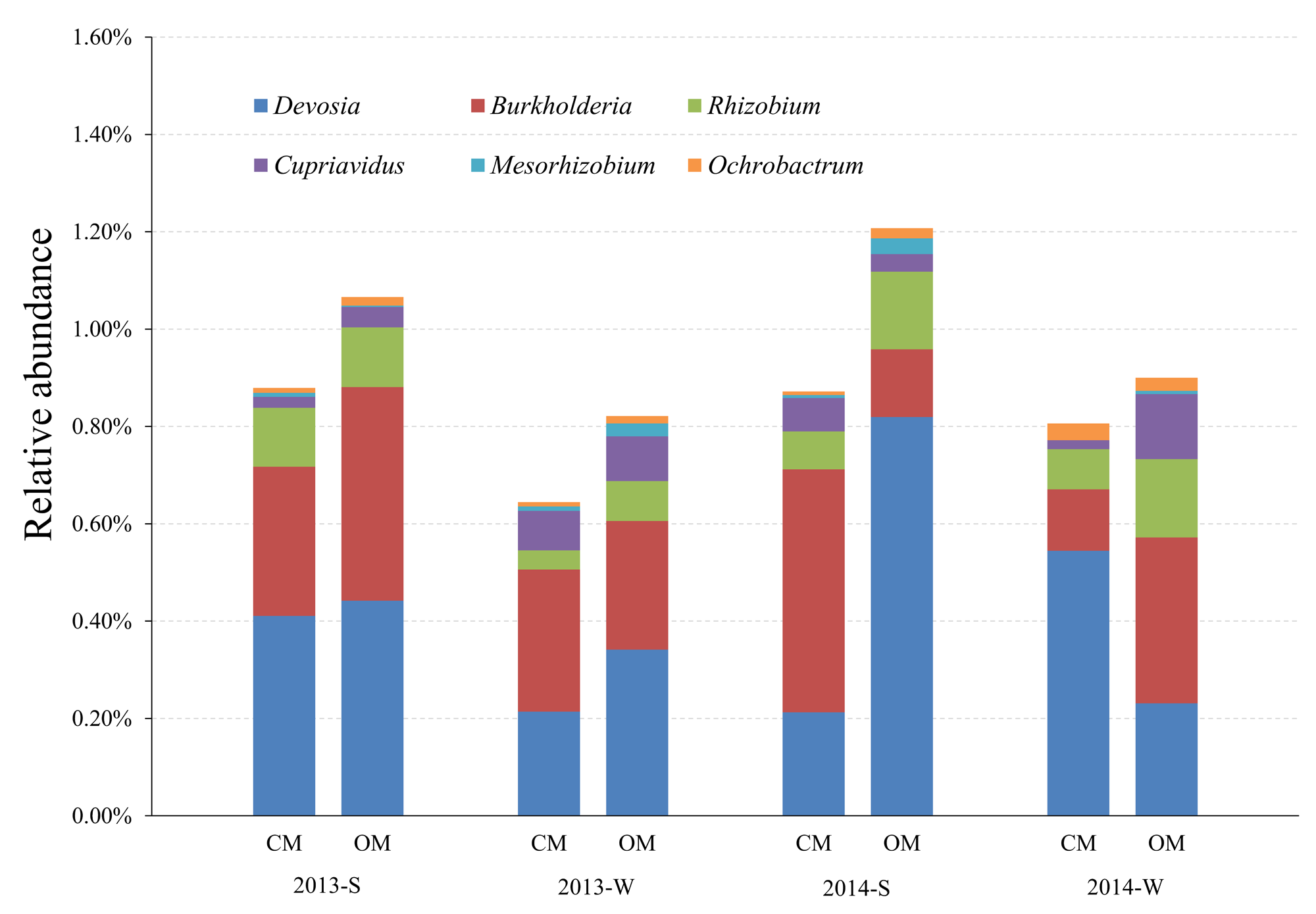

Supplement: Figure S1 — “S” for sampling time means summer at mid-June and “W” means winter at mid-December in each year. [file peerj-04-2137-s001.png]

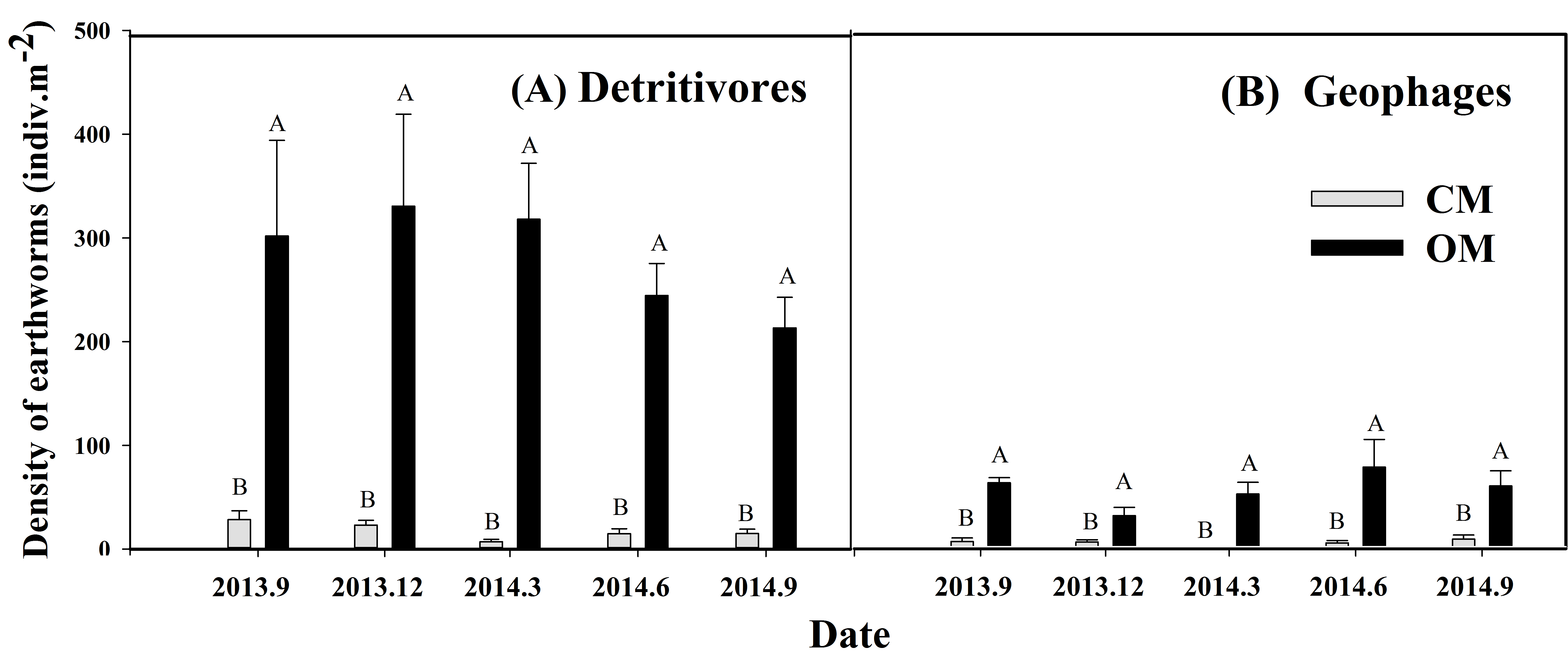

Supplement: Figure S2 — Data are means + standard error. Bars with different capital letters mean significant difference at P < 0.01 level (Student’s t-test) between two treatments within each sampling time. [file peerj-04-2137-s002.png]
