# Supplementary material for: Biodiversity management of organic orchard enhances both ecological and economic profitability
Source: PeerJ. 2016 Jun 23;4:e2137. doi: 10.7717/peerj.2137 (PMC4924131; doi:10.7717/peerj.2137)
Supplement: Table S1 [file peerj-04-2137-s003.docx]

**Table S1:** Barcode sequences used for respective soil samples of organic management (OM) and conventional management (CM). Each management had three replicates.

| **Sample ID** | **Barcode Sequence** |
| --- | --- |
| CM-2013S1 | ACAGCAGA,GATGAATC |
| CM -2013S2 | TTCACGCACTA,GTCGTAGACGT |
| CM -2013S3 | ACAGCAGA,GCCACATATC |
| OM-2013S1 | TTCACGCACTA,GATGAATC |
| OM-2013S2 | CGAACTTAC,GTCGTAGACGT |
| OM-2013S3 | CGCATACAAC,GAGTTAGCGTCAC |
| CM -2013W1 | CTGGCATAGA,GATGAATC |
| CM -2013W2 | CTGGCATAGA,AACGTGATC |
| CM -2013W3 | CTGGCATAGA,GCCACATATC |
| OM-2013W1 | CTGGCATAGA,GTCGTAGACGT |
| OM-2013W2 | CTGGCATAGA,CCTCTATCACGT |
| OM-2013W3 | CTGGCATAGA,GAGTTAGCGTCAC |
| CM -2014S1 | ACAGCAGA,AACGTGATC |
| CM -2014S2 | TTCACGCACTA,CCTCTATCACGT |
| CM -2014S3 | ACAGCAGA,GAGTTAGCGTCAC |
| OM-2014S1 | TTCACGCACTA,AACGTGATC |
| OM-2014S2 | CGAACTTAC,CCTCTATCACGT |
| OM-2014S3 | TTCACGCACTA,GCCACATATC |
| CM -2014W1 | GACTAGTAT,AACGTGATC |
| CM -2014W2 | ATAGCGACGTACA,CCTCTATCACGT |
| CM -2014W3 | CGCATACAAC,AACGTGATC |
| OM-2014W1 | ACAGCAGA,CCTCTATCACGT |
| OM-2014W2 | ATAGCGACGTACA,AACGTGATC |
| OM-2014W3 | GACTAGTAT,GAGTTAGCGTCAC |
