# Supplementary material for: Biodiversity management of organic orchard enhances both ecological and economic profitability
Source: PeerJ. 2016 Jun 23;4:e2137. doi: 10.7717/peerj.2137 (PMC4924131; doi:10.7717/peerj.2137)
Supplement: Table S2 — LC is short for Liquid chromatography; GC is short for Gas chromatography. All of the pesticides residues were less than the limit of detection which met the EU’s organic food standards. [file peerj-04-2137-s004.docx]

**Table S2:** Detection results of 191 kinds of chemical pesticides and herbicides residues in apple fruits of organic management (OM) and conventional management (CM) (Unit: mg kg^-1^).

| **Analytes** | **Methods** | **Results** | |
| --- | --- | --- | --- |
|  |  | **CM** | **OM** |
| 2-phenyl-phenol | EURL-FV (2010-M4) SA/SOP/SUM/304GC V4.0 | < 0.01 | < 0.01 |
| Acephate | EURL-FV (2010-M4) SA/SOP/SUM/304LC V3.0 | < 0.01 | < 0.01 |
| Acetamiprid | EURL-FV (2010-M4) SA/SOP/SUM/304LC V3.0 | < 0.01 | < 0.01 |
| Acetochlor | EURL-FV (2010-M4) SA/SOP/SUM/304GC V4.0 | < 0.01 | < 0.01 |
| Aldicarb | EURL-FV (2010-M4) SA/SOP/SUM/304LC V3.0 | < 0.01 | < 0.01 |
| Aldicarb-sulfone/Aldoxycarb | EURL-FV (2010-M4) SA/SOP/SUM/304LC V3.0 | < 0.01 | < 0.01 |
| Aldicarb-sulfoxide | EURL-FV (2010-M4) SA/SOP/SUM/304LC V3.0 | < 0.01 | < 0.01 |
| Atrazine | EURL-FV (2010-M4) SA/SOP/SUM/304GC V4.0 | < 0.01 | < 0.01 |
| Azinphos-methyl | EURL-FV (2010-M4) SA/SOP/SUM/304LC V3.0 | < 0.01 | < 0.01 |
| Azoxystrobin | EURL-FV (2010-M4) SA/SOP/SUM/304LC V3.0 | < 0.01 | < 0.01 |
| Benalaxyl & Benalaxyl-M | EURL-FV (2010-M4) SA/SOP/SUM/304LC V3.0 | < 0.01 | < 0.01 |
| Bendiocarb | EURL-FV (2010-M4) SA/SOP/SUM/304GC V4.0 | < 0.01 | < 0.01 |
| Benfluralin | EURL-FV (2010-M4) SA/SOP/SUM/304GC V4.0 | < 0.01 | < 0.01 |
| Benfuracarb | EURL-FV (2010-M4) SA/SOP/SUM/304LC V3.0 | < 0.01 | < 0.01 |
| Benoxacor | EURL-FV (2010-M4) SA/SOP/SUM/304GC V4.0 | < 0.01 | < 0.01 |
| Bensulfuron-methyl | EURL-FV (2010-M4) SA/SOP/SUM/304LC V3.0 | < 0.01 | < 0.01 |
| Bifenthrin | EURL-FV (2010-M4) SA/SOP/SUM/304GC V4.0 | < 0.01 | < 0.01 |
| Boscalid | EURL-FV (2010-M4) SA/SOP/SUM/304LC V3.0 | < 0.01 | < 0.01 |
| Bromopropylate | EURL-FV (2010-M4) SA/SOP/SUM/304GC V4.0 | < 0.01 | < 0.01 |
| Bupirimate | EURL-FV (2010-M4) SA/SOP/SUM/304LC V3.0 | < 0.01 | < 0.01 |
| Buprofezin | EURL-FV (2010-M4) SA/SOP/SUM/304GC V4.0 | < 0.01 | < 0.01 |
| Butachlor | EURL-FV (2010-M4) SA/SOP/SUM/304GC V4.0 | < 0.01 | < 0.01 |
| Butocarboxim | EURL-FV (2010-M4) SA/SOP/SUM/304LC V3.0 | < 0.01 | < 0.01 |
| Cadusafos | EURL-FV (2010-M4) SA/SOP/SUM/304GC V4.0 | < 0.01 | < 0.01 |
| Captan | EURL-FV (2010-M4) SA/SOP/SUM/304GC V4.0 | < 0.01 | < 0.01 |
| Carbaryl | EURL-FV (2010-M4) SA/SOP/SUM/304LC V3.0 | < 0.01 | < 0.01 |
| Carbendazim | EURL-FV (2010-M4) SA/SOP/SUM/304LC V3.0 | < 0.01 | < 0.01 |
| Carbofuran | EURL-FV (2010-M4) SA/SOP/SUM/304LC V3.0 | < 0.01 | < 0.01 |
| Carbofuran-3-hydroxy | EURL-FV (2010-M4) SA/SOP/SUM/304LC V3.0 | < 0.01 | < 0.01 |
| Carbosulfan | EURL-FV (2010-M4) SA/SOP/SUM/304LC V3.0 | < 0.05 | < 0.05 |
| Chlorbenzuron | EURL-FV (2010-M4) SA/SOP/SUM/304LC V3.0 | 0.14 | < 0.01 |
| Chlordane | EURL-FV (2010-M4) SA/SOP/SUM/304GC V4.0 | < 0.01 | < 0.01 |
| Chlorfenapyr | EURL-FV (2010-M4) SA/SOP/SUM/304GC V4.0 | < 0.01 | < 0.01 |
| Chlorfenvinphos | EURL-FV (2010-M4) SA/SOP/SUM/304GC V4.0 | < 0.01 | < 0.01 |
| Chlorpropham | EURL-FV (2010-M4) SA/SOP/SUM/304GC V4.0 | < 0.01 | < 0.01 |
| Chlorpyrifos | EURL-FV (2010-M4) SA/SOP/SUM/304GC V4.0 | 0.01 | < 0.01 |
| Chlorpyrifos Methyl | EURL-FV (2010-M4) SA/SOP/SUM/304GC V4.0 | < 0.01 | < 0.01 |
| Clethodim | EURL-FV (2010-M4) SA/SOP/SUM/304LC V3.0 | < 0.01 | < 0.01 |
| Clothianidin | EURL-FV (2010-M4) SA/SOP/SUM/304LC V3.0 | < 0.01 | < 0.01 |
| Cyanazine | EURL-FV (2010-M4) SA/SOP/SUM/304LC V3.0 | < 0.01 | < 0.01 |
| Cyflufenamid | EURL-FV (2010-M4) SA/SOP/SUM/304GC V4.0 | < 0.01 | < 0.01 |
| Cyfluthrin | EURL-FV (2010-M4) SA/SOP/SUM/304GC V4.0 | < 0.01 | < 0.01 |
| lambda-Cyhalothrin | EURL-FV (2010-M4) SA/SOP/SUM/304GC V4.0 | < 0.01 | < 0.01 |
| Cymoxanil | EURL-FV (2010-M4) SA/SOP/SUM/304LC V3.0 | < 0.01 | < 0.01 |
| Cypermethrin & zeta-Cypermethrin | EURL-FV (2010-M4) SA/SOP/SUM/304GC V4.0 | < 0.01 | < 0.01 |
| Cyprodinil | EURL-FV (2010-M4) SA/SOP/SUM/304LC V3.0 | < 0.01 | < 0.01 |
| Cyromazine | EURL-FV (2010-M4) SA/SOP/SUM/304LC V3.0 | < 0.01 | < 0.01 |
| DDD(o,p') | EURL-FV (2010-M4) SA/SOP/SUM/304GC V4.0 | < 0.01 | < 0.01 |
| DDD(p,p') | EURL-FV (2010-M4) SA/SOP/SUM/304GC V4.0 | < 0.01 | < 0.01 |
| DDE(o,p') | EURL-FV (2010-M4) SA/SOP/SUM/304GC V4.0 | < 0.01 | < 0.01 |
| DDE(p,p') | EURL-FV (2010-M4) SA/SOP/SUM/304GC V4.0 | < 0.01 | < 0.01 |
| DDT(o,p') | EURL-FV (2010-M4) SA/SOP/SUM/304GC V4.0 | < 0.01 | < 0.01 |
| DDT(p,p') | EURL-FV (2010-M4) SA/SOP/SUM/304GC V4.0 | < 0.01 | < 0.01 |
| Deltamethrin & Tralomethrin | EURL-FV (2010-M4) SA/SOP/SUM/304GC V4.0 | < 0.01 | < 0.01 |
| Diazinon | EURL-FV (2010-M4) SA/SOP/SUM/304GC V4.0 | < 0.01 | < 0.01 |
| Dichlofluanid | EURL-FV (2010-M4) SA/SOP/SUM/304GC V4.0 | < 0.01 | < 0.01 |
| Dichlorvos | EURL-FV (2010-M4) SA/SOP/SUM/304GC V4.0 | < 0.01 | < 0.01 |
| Dicloran | EURL-FV (2010-M4) SA/SOP/SUM/304GC V4.0 | < 0.01 | < 0.01 |
| Dicofol | EURL-FV (2010-M4) SA/SOP/SUM/304GC V4.0 | < 0.01 | < 0.01 |
| Diethofencarb | EURL-FV (2010-M4) SA/SOP/SUM/304LC V3.0 | < 0.01 | < 0.01 |
| Difenoconazole | EURL-FV (2010-M4) SA/SOP/SUM/304LC V3.0 | < 0.01 | < 0.01 |
| Dimethoate | EURL-FV (2010-M4) SA/SOP/SUM/304LC V3.0 | < 0.01 | < 0.01 |
| Dimethomorph | EURL-FV (2010-M4) SA/SOP/SUM/304LC V3.0 | < 0.01 | < 0.01 |
| Diniconazole | EURL-FV (2010-M4) SA/SOP/SUM/304LC V3.0 | < 0.01 | < 0.01 |
| Edifenphos | EURL-FV (2010-M4) SA/SOP/SUM/304LC V3.0 | < 0.01 | < 0.01 |
| Emamectin benzoate | EURL-FV (2010-M4) SA/SOP/SUM/304LC V3.0 | < 0.01 | < 0.01 |
| beta-endosulfan | EURL-FV (2010-M4) SA/SOP/SUM/304GC V4.0 | < 0.01 | < 0.01 |
| Endosulfan sulfate | EURL-FV (2010-M4) SA/SOP/SUM/304GC V4.0 | < 0.01 | < 0.01 |
| alpha-endosulfan | EURL-FV (2010-M4) SA/SOP/SUM/304GC V4.0 | < 0.01 | < 0.01 |
| Ethiofencarb | EURL-FV (2010-M4) SA/SOP/SUM/304LC V3.0 | < 0.01 | < 0.01 |
| Ethion | EURL-FV (2010-M4) SA/SOP/SUM/304GC V4.0 | < 0.01 | < 0.01 |
| Ethoprophos | EURL-FV (2010-M4) SA/SOP/SUM/304GC V4.0 | < 0.01 | < 0.01 |
| Etofenprox | EURL-FV (2010-M4) SA/SOP/SUM/304GC V4.0 | < 0.01 | < 0.01 |
| Etrimfos | EURL-FV (2010-M4) SA/SOP/SUM/304GC V4.0 | < 0.01 | < 0.01 |
| Famoxadone | EURL-FV (2010-M4) SA/SOP/SUM/304GC V4.0 | < 0.01 | < 0.01 |
| Fenarimol | EURL-FV (2010-M4) SA/SOP/SUM/304GC V4.0 | < 0.01 | < 0.01 |
| Fenhexamid | EURL-FV (2010-M4) SA/SOP/SUM/304LC V3.0 | < 0.01 | < 0.01 |
| Fenitrothion | EURL-FV (2010-M4) SA/SOP/SUM/304GC V4.0 | < 0.01 | < 0.01 |
| Fenobucarb | EURL-FV (2010-M4) SA/SOP/SUM/304GC V4.0 | < 0.01 | < 0.01 |
| Fenoxycarb | EURL-FV (2010-M4) SA/SOP/SUM/304LC V3.0 | < 0.01 | < 0.01 |
| Fenpropathrin | EURL-FV (2010-M4) SA/SOP/SUM/304GC V4.0 | < 0.01 | < 0.01 |
| Fenpropimorph | EURL-FV (2010-M4) SA/SOP/SUM/304LC V3.0 | < 0.01 | < 0.01 |
| Fenpyroximate | EURL-FV (2010-M4) SA/SOP/SUM/304LC V3.0 | < 0.01 | < 0.01 |
| Fenthion | EURL-FV (2010-M4) SA/SOP/SUM/304GC V4.0 | < 0.01 | < 0.01 |
| Fenvalerate & Esfenvalerate | EURL-FV (2010-M4) SA/SOP/SUM/304GC V4.0 | < 0.01 | < 0.01 |
| Fipronil | EURL-FV (2010-M4) SA/SOP/SUM/304GC V4.0 | < 0.01 | < 0.01 |
| Fluazifop-butyl & Fluazifop-p butyl | EURL-FV (2010-M4) SA/SOP/SUM/304GC V4.0 | < 0.01 | < 0.01 |
| Flucythrinate | EURL-FV (2010-M4) SA/SOP/SUM/304GC V4.0 | < 0.01 | < 0.01 |
| Flufenoxuron | EURL-FV (2010-M4) SA/SOP/SUM/304LC V3.0 | < 0.01 | < 0.01 |
| Flusilazole | EURL-FV (2010-M4) SA/SOP/SUM/304GC V4.0 | < 0.01 | < 0.01 |
| Fluvalinate | EURL-FV (2010-M4) SA/SOP/SUM/304GC V4.0 | < 0.01 | < 0.01 |
| Furathiocarb | EURL-FV (2010-M4) SA/SOP/SUM/304LC V3.0 | < 0.01 | < 0.01 |
| gamma-HCH(gamma-BHC or Lindane) | EURL-FV (2010-M4) SA/SOP/SUM/304GC V4.0 | < 0.01 | < 0.01 |
| Heptenophos | EURL-FV (2010-M4) SA/SOP/SUM/304GC V4.0 | < 0.01 | < 0.01 |
| Hexythiazox | EURL-FV (2010-M4) SA/SOP/SUM/304LC V3.0 | < 0.01 | < 0.01 |
| Imazalil | EURL-FV (2010-M4) SA/SOP/SUM/304LC V3.0 | < 0.01 | < 0.01 |
| Imidacloprid | EURL-FV (2010-M4) SA/SOP/SUM/304LC V3.0 | < 0.01 | < 0.01 |
| Indoxacarb | EURL-FV (2010-M4) SA/SOP/SUM/304LC V3.0 | < 0.01 | < 0.01 |
| Iprodione | EURL-FV (2010-M4) SA/SOP/SUM/304GC V4.0 | < 0.01 | < 0.01 |
| Iprovalicarb | EURL-FV (2010-M4) SA/SOP/SUM/304LC V3.0 | < 0.01 | < 0.01 |
| Isocarbophos | EURL-FV (2010-M4) SA/SOP/SUM/304LC V3.0 | < 0.01 | < 0.01 |
| Isofenphos | EURL-FV (2010-M4) SA/SOP/SUM/304GC V4.0 | < 0.01 | < 0.01 |
| Isofenphos-methyl | EURL-FV (2010-M4) SA/SOP/SUM/304GC V4.0 | < 0.01 | < 0.01 |
| Isoprocarb | EURL-FV (2010-M4) SA/SOP/SUM/304GC V4.0 | < 0.01 | < 0.01 |
| Isoprothiolane | EURL-FV (2010-M4) SA/SOP/SUM/304GC V4.0 | < 0.01 | < 0.01 |
| Isoproturon | EURL-FV (2010-M4) SA/SOP/SUM/304LC V3.0 | < 0.01 | < 0.01 |
| Kresoxim-methyl | EURL-FV (2010-M4) SA/SOP/SUM/304GC V4.0 | < 0.01 | < 0.01 |
| Linuron | EURL-FV (2010-M4) SA/SOP/SUM/304LC V3.0 | < 0.01 | < 0.01 |
| Malathion | EURL-FV (2010-M4) SA/SOP/SUM/304GC V4.0 | < 0.01 | < 0.01 |
| Metalaxyl&Mefenoxam | EURL-FV (2010-M4) SA/SOP/SUM/304GC V4.0 | < 0.01 | < 0.01 |
| Metamitron | EURL-FV (2010-M4) SA/SOP/SUM/304LC V3.0 | < 0.01 | < 0.01 |
| Methamidophos | EURL-FV (2010-M4) SA/SOP/SUM/304LC V3.0 | < 0.01 | < 0.01 |
| Methidathion | EURL-FV (2010-M4) SA/SOP/SUM/304GC V4.0 | < 0.01 | < 0.01 |
| Methiocarb | EURL-FV (2010-M4) SA/SOP/SUM/304LC V3.0 | < 0.01 | < 0.01 |
| Methomyl | EURL-FV (2010-M4) SA/SOP/SUM/304LC V3.0 | < 0.01 | < 0.01 |
| Methoxyfenozide | EURL-FV (2010-M4) SA/SOP/SUM/304LC V3.0 | < 0.01 | < 0.01 |
| Metolachlor&S-Metolachlor | EURL-FV (2010-M4) SA/SOP/SUM/304LC V3.0 | < 0.01 | < 0.01 |
| Mevinphos | EURL-FV (2010-M4) SA/SOP/SUM/304GC V4.0 | < 0.01 | < 0.01 |
| Monocrotophos | EURL-FV (2010-M4) SA/SOP/SUM/304LC V3.0 | < 0.01 | < 0.01 |
| Myclobutanil | EURL-FV (2010-M4) SA/SOP/SUM/304GC V4.0 | < 0.01 | < 0.01 |
| Napropamide | EURL-FV (2010-M4) SA/SOP/SUM/304GC V4.0 | < 0.01 | < 0.01 |
| Nicosulfuron | EURL-FV (2010-M4) SA/SOP/SUM/304LC V3.0 | < 0.01 | < 0.01 |
| Nitrothal-isopropyl | EURL-FV (2010-M4) SA/SOP/SUM/304GC V4.0 | < 0.01 | < 0.01 |
| Octachlorodipropyl ether | EURL-FV (2010-M4) SA/SOP/SUM/304LC V3.0 | < 0.01 | < 0.01 |
| Omethoate | EURL-FV (2010-M4) SA/SOP/SUM/304LC V3.0 | < 0.01 | < 0.01 |
| Oxadiazon | EURL-FV (2010-M4) SA/SOP/SUM/304GC V4.0 | < 0.01 | < 0.01 |
| Oxadixyl | EURL-FV (2010-M4) SA/SOP/SUM/304LC V3.0 | < 0.01 | < 0.01 |
| Oxydemeton-methyl | EURL-FV (2010-M4) SA/SOP/SUM/304GC V4.0 | < 0.01 | < 0.01 |
| Paclobutrazol | EURL-FV (2010-M4) SA/SOP/SUM/304GC V4.0 | < 0.01 | < 0.01 |
| Parathion | EURL-FV (2010-M4) SA/SOP/SUM/304GC V4.0 | < 0.01 | < 0.01 |
| Parathion-methyl | EURL-FV (2010-M4) SA/SOP/SUM/304GC V4.0 | < 0.01 | < 0.01 |
| Penconazole | EURL-FV (2010-M4) SA/SOP/SUM/304GC V4.0 | < 0.01 | < 0.01 |
| Pendimethalin | EURL-FV (2010-M4) SA/SOP/SUM/304GC V4.0 | < 0.01 | < 0.01 |
| Permethrin | EURL-FV (2010-M4) SA/SOP/SUM/304GC V4.0 | < 0.01 | < 0.01 |
| Phenthoate | EURL-FV (2010-M4) SA/SOP/SUM/304GC V4.0 | < 0.01 | < 0.01 |
| Phorate | EURL-FV (2010-M4) SA/SOP/SUM/304LC V3.0 | < 0.01 | < 0.01 |
| Phorate-sulfone | EURL-FV (2010-M4) SA/SOP/SUM/304LC V3.0 | < 0.01 | < 0.01 |
| Phorate-sulfoxide | EURL-FV (2010-M4) SA/SOP/SUM/304GC V4.0 | < 0.01 | < 0.01 |
| Phosalone | EURL-FV (2010-M4) SA/SOP/SUM/304GC V4.0 | < 0.01 | < 0.01 |
| Phosmet | EURL-FV (2010-M4) SA/SOP/SUM/304LC V3.0 | < 0.01 | < 0.01 |
| Phosphamidone | EURL-FV (2010-M4) SA/SOP/SUM/304LC V3.0 | < 0.01 | < 0.01 |
| Phoxim | EURL-FV (2010-M4) SA/SOP/SUM/304LC V3.0 | < 0.01 | < 0.01 |
| Pirimicarb | EURL-FV (2010-M4) SA/SOP/SUM/304GC V4.0 | < 0.01 | < 0.01 |
| Pirimiphos-ethyl | EURL-FV (2010-M4) SA/SOP/SUM/304GC V4.0 | < 0.01 | < 0.01 |
| Pirimiphos-methyl | EURL-FV (2010-M4) SA/SOP/SUM/304LC V3.0 | < 0.01 | < 0.01 |
| Prochloraz | EURL-FV (2010-M4) SA/SOP/SUM/304GC V4.0 | < 0.01 | < 0.01 |
| Procymidone | EURL-FV (2010-M4) SA/SOP/SUM/304GC V4.0 | < 0.01 | < 0.01 |
| Profenofos | EURL-FV (2010-M4) SA/SOP/SUM/304LC V3.0 | < 0.01 | < 0.01 |
| Promecarb | EURL-FV (2010-M4) SA/SOP/SUM/304GC V4.0 | < 0.01 | < 0.01 |
| Prometryn | EURL-FV (2010-M4) SA/SOP/SUM/304LC V3.0 | < 0.01 | < 0.01 |
| Propamocarb | EURL-FV (2010-M4) SA/SOP/SUM/304GC V4.0 | < 0.01 | < 0.01 |
| Propargite | EURL-FV (2010-M4) SA/SOP/SUM/304GC V4.0 | < 0.01 | < 0.01 |
| Propham | EURL-FV (2010-M4) SA/SOP/SUM/304GC V4.0 | < 0.01 | < 0.01 |
| Propiconazole | EURL-FV (2010-M4) SA/SOP/SUM/304GC V4.0 | < 0.01 | < 0.01 |
| Propoxur | EURL-FV (2010-M4) SA/SOP/SUM/304GC V4.0 | < 0.01 | < 0.01 |
| Propyzamide | EURL-FV (2010-M4) SA/SOP/SUM/304LC V3.0 | < 0.01 | < 0.01 |
| Pymetrozine | EURL-FV (2010-M4) SA/SOP/SUM/304GC V4.0 | < 0.01 | < 0.01 |
| Pyrazophos | EURL-FV (2010-M4) SA/SOP/SUM/304GC V4.0 | < 0.01 | < 0.01 |
| Pyridaben | EURL-FV (2010-M4) SA/SOP/SUM/304GC V4.0 | < 0.01 | < 0.01 |
| Pyridaphenthion | EURL-FV (2010-M4) SA/SOP/SUM/304LC V3.0 | < 0.01 | < 0.01 |
| Pyrimethanil | EURL-FV (2010-M4) SA/SOP/SUM/304GC V4.0 | < 0.01 | < 0.01 |
| Quinalphos | EURL-FV (2010-M4) SA/SOP/SUM/304GC V4.0 | < 0.01 | < 0.01 |
| Quintozene | EURL-FV (2010-M4) SA/SOP/SUM/304LC V3.0 | < 0.01 | < 0.01 |
| Quizalofop-ethyl&Quizalofop-p-ethyl | EURL-FV (2010-M4) SA/SOP/SUM/304LC V3.0 | < 0.01 | < 0.01 |
| Rimsulfuron | EURL-FV (2010-M4) SA/SOP/SUM/304GC V4.0 | < 0.01 | < 0.01 |
| Simazine | EURL-FV (2010-M4) SA/SOP/SUM/304GC V4.0 | < 0.01 | < 0.01 |
| Spinosad | EURL-FV (2010-M4) SA/SOP/SUM/304LC V3.0 | < 0.01 | < 0.01 |
| Spiroxamine | EURL-FV (2010-M4) SA/SOP/SUM/304LC V3.0 | < 0.01 | < 0.01 |
| Tebuconazole | EURL-FV (2010-M4) SA/SOP/SUM/304GC V4.0 | 0.01 | < 0.01 |
| Tebufenozide | EURL-FV (2010-M4) SA/SOP/SUM/304LC V3.0 | < 0.01 | < 0.01 |
| Tetrachlorvinphos | EURL-FV (2010-M4) SA/SOP/SUM/304GC V4.0 | < 0.01 | < 0.01 |
| Tetradifon | EURL-FV (2010-M4) SA/SOP/SUM/304GC V4.0 | < 0.01 | < 0.01 |
| Thiabendazole | EURL-FV (2010-M4) SA/SOP/SUM/304LC V3.0 | < 0.01 | < 0.01 |
| Thiacloprid | EURL-FV (2010-M4) SA/SOP/SUM/304LC V3.0 | < 0.01 | < 0.01 |
| Thiamethoxam | EURL-FV (2010-M4) SA/SOP/SUM/304GC V4.0 | < 0.01 | < 0.01 |
| Thifensulfuron-methyl | EURL-FV (2010-M4) SA/SOP/SUM/304LC V3.0 | < 0.01 | < 0.01 |
| Thiodicarb | EURL-FV (2010-M4) SA/SOP/SUM/304LC V3.0 | < 0.01 | < 0.01 |
| Thiofanox-sulfone | EURL-FV (2010-M4) SA/SOP/SUM/304LC V3.0 | < 0.01 | < 0.01 |
| Thiofanox-sulfoxide | EURL-FV (2010-M4) SA/SOP/SUM/304LC V3.0 | < 0.01 | < 0.01 |
| Tolclofos-methyl | EURL-FV (2010-M4) SA/SOP/SUM/304GC V4.0 | < 0.01 | < 0.01 |
| Tolylfluanid | EURL-FV (2010-M4) SA/SOP/SUM/304GC V4.0 | < 0.01 | < 0.01 |
| Triadimefon | EURL-FV (2010-M4) SA/SOP/SUM/304GC V4.0 | < 0.01 | < 0.01 |
| Triadimenol | EURL-FV (2010-M4) SA/SOP/SUM/304GC V4.0 | < 0.01 | < 0.01 |
| Triasulfuron | EURL-FV (2010-M4) SA/SOP/SUM/304LC V3.0 | < 0.01 | < 0.01 |
| Triazophos | EURL-FV (2010-M4) SA/SOP/SUM/304GC V4.0 | < 0.01 | < 0.01 |
| Trichlorfon | EURL-FV (2010-M4) SA/SOP/SUM/304LC V3.0 | < 0.01 | < 0.01 |
| Triflumizole | EURL-FV (2010-M4) SA/SOP/SUM/304LC V3.0 | < 0.01 | < 0.01 |
| Trifluralin | EURL-FV (2010-M4) SA/SOP/SUM/304GC V4.0 | < 0.01 | < 0.01 |
| Triflusulfuron-methyl | EURL-FV (2010-M4) SA/SOP/SUM/304LC V3.0 | < 0.01 | < 0.01 |
| Vamidothion | EURL-FV (2010-M4) SA/SOP/SUM/304LC V3.0 | < 0.01 | < 0.01 |
| Vinclozolin | EURL-FV (2010-M4) SA/SOP/SUM/304GC V4.0 | < 0.01 | < 0.01 |

Notes: LC is short for Liquid chromatography; GC is short for Gas chromatography.

All of the pesticides residues were less than the limit of detection which met the EU’s organic food standards.
