# Supplementary material for: Biodiversity management of organic orchard enhances both ecological and economic profitability
Source: PeerJ. 2016 Jun 23;4:e2137. doi: 10.7717/peerj.2137 (PMC4924131; doi:10.7717/peerj.2137)
Supplement: Table S3 — Apples were divided into six grades according to the marketable price, which was decided by the weight and appearance of the fruits. Total output was the sum of all the output in each class, which was calculated through yield of each class multiply unit price. Average annual output was the average of the output of the three years. Output/input ratio was calculate through dividing annual average output by annual average input. [file peerj-04-2137-s005.docx]

**Table S3:** Details of analysis on inputs and outputs of organic management (OM) and conventional management (CM).

| **CM** | | | | | | **OM** | | | | |
| --- | --- | --- | --- | --- | --- | --- | --- | --- | --- | --- |
| **Input** | | | | | | | | | | |
| **Practice** | **Details** | | | **($ ha^-1^)** | | **Details** | | | **($ ha^-1^)** | |
| **Fertilization** | Urea:1,500 kg*0.39 $ kg^-1^ | | | 581.04 | | Cattle manure: 217.5 cube ha^-1^*4.84 $ cube^-1^ | | | 1053.00 | |
|  | Potassium sulphate compound fertilizer:  5,310 kg*0.48 $ kg^-1^ | | | 2571.10 | |  |  |  |  |  |
| **Pest & disease  control** | Mar. Lime sulfur | | | 113.50 | | Biogas slurry: 36.3 $ per time, 3 times | | | 108.95 | |
|  | Imidacloprid | | | 20.05 | | Lime sulfur | | | 113.50 | |
|  | Beta-cypermethrin | | | 13.80 | | Bordeaux mixture: 115 $ per time, 3 times | | | 344.99 | |
|  | Mancozeb | | | 87.64 | | Sticky card | | | 145.26 | |
|  | Thiophanate methyl | | | 93.06 | | Stem residue trap | | | 169.47 | |
|  | May Chlorpyrifos | | | 30.70 | | Solar energy light-traps depreciation costs (5 years), 322.8 $ light^-1^, 9 lights ha^-1^ | | | 581 | |
|  | Hexythiazox | | | 27.67 | |  | | |  | |
|  | Mannitol chelating calcium | | | 34.86 | |  | | |  | |
|  | Propineb+tebuconazole | | | 58.10 | |  | | |  | |
|  | Carbendazim | | | 34.86 | |  | | |  | |
|  | Mancozeb | | | 87.64 | |  | | |  | |
|  | Jun. Azacyclotin | | | 36.32 | |  | | |  | |
|  | Imidacloprid | | | 20.05 | |  | | |  | |
|  | Chlorbenzuron | | | 23.24 | |  | | |  | |
|  | Jul. Chlorbenzuron | | | 23.24 | |  | | |  | |
|  | Hexythiazox | | | 27.67 | |  | | |  | |
|  | Beta-cypermethrin | | | 13.80 | |  | | |  | |
|  | Carbendazim | | | 34.86 | |  | | |  | |
|  | Aug. Chlorbenzuron | | | 23.24 | |  | | |  | |
|  | Hexythiazox | | | 27.67 | |  | | |  | |
|  | Beta-cypermethrin | | | 13.80 | |  | | |  | |
|  | Mancozeb | | | 87.64 | |  | | |  | |
|  | Sep. Chlorbenzuron | | | 23.24 | |  | | |  | |
|  | Hexythiazox | | | 27.67 | |  | | |  | |
|  | Beta-cypermethrin | | | 13.80 | |  | | |  | |
|  | Carbendazim | | | 34.86 | |  | | |  | |
| **Weed control** | Paraquat emulsifier 24.21 $ labor^-1^ *3times | | | 72.63 | |  | | |  | |
| **Irrigation** | Water cost: 217.89 $ ha^-1^*4 times | | | 872.00 | | Water cost: 217.89 $ ha^-1^*4 times | | | 872.00 | |
| **Electricity** | Sprayer (pesticides): 960 KWh*0.1$ KWh^-1^ | | | 92.97 | | Sprayer | | | 40.70 | |
|  | Sprayer (herbicides): 90 KWh*0.1$ KWh^-1^ | | | 9.00 | | Irrigation | | | 26.10 | |
|  | Irrigation (750W): 270KWh*0.1$ KWh^-1^ | | | 27.00 | |  | | |  | |
| **Labor** | Scraping old bark:  9.68 $ labor^-1^*7.5 labor ha^-1^ | | | 72.63 | | Fertilization: 9.68 $ labor^-1^ * 165 labor ha^-1^ | | | 1597.86 | |
|  |  |  |  |  |  | Pest control: 135 labor ha^-1^ | | | 1307.00 | |
|  | Pruning: 30 labor ha^-1^ | | | 290.52 | | Planting *Duchesnea indica*: 7.5 labor ha^-1^ | | | 72.63 | |
|  | Clean up the ground: 15 labor ha^-1^ | | | 145.26 | | Mowing: 30 labor ha^-1^ | | | 290.52 | |
|  | Hand pollination: 30 labor ha^-1^ | | | 290.52 | | Scrape old bark: 7.5 labor ha^-1^ | | | 72.63 | |
|  | Bagging: 90 labor ha^-1^ | | | 871.56 | | Pruning: 30 labor ha^-1^ | | | 290.52 | |
|  | Bag removing: 37.5 labor ha^-1^ | | | 363.15 | | Clean up the ground: 15 labor ha^-1^ | | | 145.26 | |
|  | Blossom and fruit thinning: 75 labor ha^-1^ | | | 726.30 | | Pollination ha^-1^ | | | 0.00 | |
|  | Irrigation: 15 labor ha^-1^ | | | 145.26 | | Bagging: 75 labor ha^-1^ | | | 726.30 | |
|  | Harvest: 150 labor ha^-1^ | | | 1452.60 | | Bag removing: 30 labor ha^-1^ | | | 290.52 | |
|  | Pest & disease control: 240 labor ha^-1^ | | | 2324.16 | | Blossom and fruit thinning: 60 labor ha^-1^ | | | 581.04 | |
|  | Fertilization: 45 labor ha^-1^ | | | 435.78 | | Irrigation: 15 labor ha^-1^ | | | 145.26 | |
|  | Weed control: 11.25 labor ha^-1^ | | | 108.95 | | Harvest: 120 labor ha^-1^ | | | 1162.08 | |
| **Materials &  depreciation  costs** | Spray depreciation costs | | | 55.48 | | Mower depreciation costs | | | 69.6 | |
|  | Water pump depreciation costs | | | 34.80 | | Spray depreciation costs | | | 55.5 | |
|  | Plastic fruit bags | | | 127.10 | | Water pump depreciation costs | | | 34.8 | |
|  |  | | |  | | Apple paper bags | | | 544.7 | |
|  |  | | |  | | The bee reed pipe | | | 24.2 | |
|  |  | | |  | | Gasoline for mower | | | 84.7 | |
| **Transportation** |  | | |  | | for Cattle manure: 1.61 $ car^-1^, 150 car ha^-1^ | | | 242.1 | |
|  |  | | |  | | for Biogas slurry: 12.9 $ car^-1^, 9 car ha^-1^ | | | 116.2 | |
| **Annual average input** | 12703 | | | | | 11309 | | | | |
| **Output** | | | | | | | | | | |
| Grades | Unit price  ($ kg^-1^) | Yield (kg ha^-1^) | | | | Unit price  ($ kg^-1^) | Yield (kg ha^-1^) | | | |
|  |  | **2012** | **2013** | | **2014** |  | **2012** | **2013** | | **2014** |
| Ⅰ | 0.97 | 1613 | 1428 | | 1374 | 4.84 | 1343 | 1425 | | 1809 |
| Ⅱ | 0.81 | 11288 | 14280 | | 12366 | 3.87 | 2685 | 3705 | | 3618 |
| Ⅲ | 0.65 | 12900 | 12852 | | 12023 | 2.58 | 5370 | 5985 | | 6030 |
| Ⅳ | 0.48 | 4838 | 4998 | | 7214 | 0.48 | 9398 | 8550 | | 9347 |
| Ⅴ | 0.32 | 968 | 1428 | | 859 | 0.32 | 6713 | 7695 | | 7839 |
| Ⅵ | 0.1 | 645 | 714 | | 515 | 0.10 | 1343 | 1140 | | 1508 |
| **Average price** | 0.56 |  |  | |  | 2.03 |  |  | |  |
| **Total yield** |  | 32250 | 35700 | | 34350 |  | 26850 | 28500 | | 30150 |
| **Annual average output** | 22991 | | | | | 41521 | | | | |
| **Output/input  ratio** | 1.81 | | | | | 3.67 | | | | |

Notes: Apples were divided into six grades according to the marketable price, which was decided by the weight and appearance of the fruits. Total output was the sum of all the output in each class, which was calculated through yield of each class multiply unit price. Average annual output was the average of the output of the three years. Output/input ratio was calculate through dividing annual average output by annual average input.
